# Time-regulated transcripts with the potential to modulate human pluripotent stem cell-derived cardiomyocyte differentiation

**DOI:** 10.1186/s13287-022-03138-x

**Published:** 2022-09-02

**Authors:** Juan J. A. M. Muñoz, Rafael Dariolli, Caio Mateus da Silva, Elida A. Neri, Iuri C. Valadão, Lauro Thiago Turaça, Vanessa M. Lima, Mariana Lombardi Peres de Carvalho, Mariliza R. Velho, Eric A. Sobie, Jose E. Krieger

**Affiliations:** 1 Laboratory of Genetics and Molecular Cardiology/LIM 13, Heart Institute (InCor), University of São Paulo Medical School, Avenida Dr. Eneas C. Aguiar 44, São Paulo, SP 05403-000 Brazil; 2 Present Address: Universidad Señor de Sipán, Chiclayo, Perú; 3 Department of Pharmacological Sciences, Graduate School of Biomedical Sciences, Icahn School of Medicine at Mount Sinai, New York, NY USA

**Keywords:** Cardiac differentiation, hiPSC-CM, Time-dependent regulated transcripts, miRNA

## Abstract

**Background:**

Human-induced pluripotent stem cell-derived cardiomyocytes (hiPSC-CM) are a promising disease model, even though hiPSC-CMs cultured for extended periods display an undifferentiated transcriptional landscape. MiRNA–target gene interactions contribute to fine-tuning the genetic program governing cardiac maturation and may uncover critical pathways to be targeted.

**Methods:**

We analyzed a hiPSC-CM public dataset to identify time-regulated miRNA–target gene interactions based on three logical steps of filtering. We validated this process in silico using 14 human and mouse public datasets, and further confirmed the findings by sampling seven time points over a 30-day protocol with a hiPSC-CM clone developed in our laboratory. We then added miRNA mimics from the top eight miRNAs candidates in three cell clones in two different moments of cardiac specification and maturation to assess their impact on differentiation characteristics including proliferation, sarcomere structure, contractility, and calcium handling.

**Results:**

We uncovered 324 interactions among 29 differentially expressed genes and 51 miRNAs from 20,543 transcripts through 120 days of hiPSC-CM differentiation and selected 16 genes and 25 miRNAs based on the inverse pattern of expression (Pearson *R*-values < − 0.5) and consistency in different datasets. We validated 16 inverse interactions among eight genes and 12 miRNAs (Person *R*-values < − 0.5) during hiPSC-CMs differentiation and used miRNAs mimics to verify proliferation, structural and functional features related to maturation. We also demonstrated that miR-124 affects Ca^2+^ handling altering features associated with hiPSC-CMs maturation.

**Conclusion:**

We uncovered time-regulated transcripts influencing pathways affecting cardiac differentiation/maturation axis and showed that the top-scoring miRNAs indeed affect primarily structural features highlighting their role in the hiPSC-CM maturation.

**Supplementary Information:**

The online version contains supplementary material available at 10.1186/s13287-022-03138-x.

## Introduction

Human-induced pluripotent stem cell-derived cardiomyocytes (hiPSC-CMs) are instrumental for the complete development of cardiac disease cell-modeling systems, patient-specific drug tests, and strategies to replace lost cardiomyocytes [1–3].

A growing body of evidence suggests that differentiated hiPSC-CMs are immature despite molecular, morphological, metabolic, and functional similarities with adult cardiomyocytes [4, 5]. There is a gap in the understanding of the processes governing cardiac development and the differentiation of induced pluripotent stem cells into cardiomyocytes. Success in this area may offer opportunities to target transcriptional networks associated with specific cardiac cell phenotypes and maturation stages [6–8].

In this context, microRNAs (miRNAs), a class of non-coding RNAs (18–24 nucleotides), are responsible for post-transcriptional repression of several target genes regulating multiple biological processes [9]. MiRNAs have been exploited for their contributions to embryonic development and disease states, serving as biomarkers and therapeutic agents due to their modulatory capacity [10–14]. In vitro models of cardiac differentiation provide evidence that miRNA–target gene interactions might influence cardiomyocyte maturation. For example, miR-199a influences cardiomyocytes proliferation [15], the cluster of miRs-302 and miR-1 is associated with cardiac development [16], and the family of let-7 miRNAs, are required for stem cell-derived cardiomyocytes maturation. These findings highlight miRNAs' potential to regulate multiple genes associated with complex processes including cardiac cell differentiation.

We hypothesized that using a time-dependent strategy for transcript selection based on three logical stages of filtering would improve the discrimination of small transcriptional changes over time required to uncover gene-miRNA interactions critical for the differentiation processes of hiPSC-CMs over a 30-day period. These time-regulated genes-miRNA pairs are candidates for fine-tuning cardiomyocytes differentiation. We then validated the transcriptional signatures across eleven datasets for gene expression and three human datasets for miRNA expression from Gene Expression Omnibus. Finally, we tested against a transcriptomic database experimentally generated in our laboratory and overexpressed the most promising miRNA in three different cell clones to assess their capacity to affect surrogates of the differentiation/maturation process including proliferation, structural and functional features.

## Methods

### Unsupervised statistical analysis, Pearson correlation, and expression charts

Unsupervised statistical strategies such as PCA and hierarchical clustering (Euclidean distance) analysis were performed using MATLAB 2018a tools and homemade scripts. Correlation analysis between the transcripts was performed using the Pearson test with a Kendall rank correlation matrix from the MATLAB package. The expression charts were all plotted using codes generated in MATLAB and available upon reasonable request.

### Microarray (in vitro) coding and non-coding (miRNA) sequence of selection

To obtain two lists of genes and miRNAs of interest from the Babiarz et al*.* [16] databases (gene expression and miRNA expression chips) (Universal BeadChip (Illumina)), we used the previous log2 transformed and quantile normalized data provided by the authors in the GEO database (GSE35673). Three replicates of each time point were used in this study (biological replicates). Finally, only transcripts displaying six or more expression times in all the samples were considered for the subsequent filtering steps.

### Segmental DE analysis to select transcripts from different phases of the cardiac cell differentiation

Aiming to increase power-resolution of the transcripts to better group cells from the various stages of the cardiac cell differentiation, we designed a tool for selecting transcripts of interest based on the DE transcripts. For this strategy, the transcription expression timeline was split into segments of the differentiation (days 0–7, days 10–14, and days 20–120) that considered the most prominent biological modifications in cell fate induced by in vitro cardiomyogenesis. Only transcripts fitting the ratio interval of <  = 0.8 or >  = 1.2 were considered informative. These calculations were all performed using MATLAB scripts.

### Exponential expression analysis to select transcripts with linear to exponential fit during the cardiac cell differentiation

To group cells from different stages of the cardiac cell differentiation (particularly in the phases of maturation), we developed a second tool for the selection of transcripts of interest based on their exponential expression pattern during the differentiation. At this point, only transcripts fitting a second-degree exponential equation *R* >  = 0.7 were considered as informative. These calculations were all performed using MATLAB scripts.

### Random forest classification model construction

To evaluate our filtering approach on the genes filtering and selection we used the transcriptional data from Babiarz et al. dataset [16] and trained three separate random forest algorithms to determine whether our filtering steps could have selected the most relevant genes and miRNAs to classify cardiomyocytes in their differentiation times. In these algorithms, we used the differentiation days as prediction classes for the ML model to predict and the expressions as the predictors values to train the model. The R Caret package (version 6.0–89) was used to train the models using the genes' sets and expression levels in each group after filtering. Each gene and its expression level for each differentiation day were treated as independent variables, even though the random forest model can learn interactions between the data and improve the prediction capacity. To prevent overfitting, the training set, we used tenfold cross-validation during the algorithm training. The comparison between the prediction efficiency between the models was performed using the MLeval-R package and by creating a ROC graph and calculating the AUC for each model.

### Prediction analysis for miRNA-gene targets and enrichment using transcriptomic validated interactions

The miRNAs and genes that showed altered expression were evaluated using the most widely used algorithm miRTARbase (http://mirtarbase.mbc.nctu.edu.tw/). In silico target prediction algorithms such as TargetScan (http://www.targetscan.org/), PicTar (http: //pictar.mdc-berlin.de/) and Diana.microTv.4.0 (http://diana.cslab.ece.ntua.gr/DianaTools/index.php?r=microtv4/index) were also used to filter strongly predicted interactions not yet validated experimentally. From this database, we generated a list of experimentally validated miRNAs that potentially regulated selected genes. These interactions were evaluated using I2D software (http://ophid.utoronto.ca/ophidv2.204/) for the enrichment of transcript–transcript and protein–protein interactions. We selected the strongest interactions using a confidence score provided in the software. This score describes the relative strength of the prediction for a microRNA to gene pair for the source specified [17]. To analyze and visualize the resulting network of transcripts interaction network NAViGaTOR (Network Analysis, Visualization and Graphing Toronto) (www.ophid.utoronto.ca/navigator/), we used a combination of hardware-based graphics acceleration and highly optimized layout algorithms to enable interactive visualization of large networks [18]. The methods described below address the validation steps applied to the list of interactive transcripts obtained until this point.

### Checking the transcriptomic signature (using the Babiarz dataset)

Since the Babiarz database was used to generate the lists of target transcripts for our molecular signature of cardiac differentiation, the first validation step for selected transcripts was performed against this database. Genes and miRNAs predicted to be interactive in several other biological models were plotted together. These charts were prepared by plotting time on the X-axis (days 0–120) and log2 quantile normalized expression on the Y-axis from the Babiarz dataset. The correlation between gene expression and miRNAs was tested by plotting these values in a Kendall rank correlation matrix. These calculations were performed in MATLAB. Pearson linear correlation algorithm was chosen to calculate the correlations found by the Kendall matrix. Pearson R-values and P-values were calculated and used to rank the best gene-miRNA interactions found during cardiac cell differentiation.

### In silico validation and curation of the transcriptomic signature in different databases and species

After performing the internal validation of the transcriptomic signature by tracking how these interactive transcripts behave over time, a second in silico validation was performed using other databases of cardiac cell differentiation found in the literature. The fold-change between day 0 and the final day of each selected transcript was calculated. Then, the orientation of these changes was compared with the fold-change observed for the Babiarz dataset*.* The power of each transcript was represented by the percentage of datasets transcript showing the same fold-change direction. These results were presented in an N# of observation plot and tables (Additional file 1: Table S1).

PCA and hierarchical clustering analysis were performed to demonstrate the capacity of our signature to segregate different cells during cardiac cell differentiation. Human and mouse databases were interrogated. More details about the databases used can be found in the GEO datasets details spreadsheet in Additional file 1: Table S1.

### Functional classification and comparative analysis of hiPSC-CMs and cardiac tissues expression of genes of interest

Our transcriptomic signature partners were subjected to functional classification, in separated strategies, genes, and miRNAs. The Database for Annotation, Identity and Integrated Discover identity converter (DAVID) (http://david.abcc.ncrFCrf.gov/tools.jsp) was used to update the official gene symbols. Genes were subjected to Enrichr software (http:// http://amp.pharm.mssm.edu/Enrichr/). MiRNAs were subjected to *miRSystem software* (http:// http://mirsystem.cgm.ntu.edu.tw/). The P-values and scores provided by the analysis of these programs were used to classify the main pathways and tissues in which our selected transcripts can be found. The bar charts were plotted using MATLAB homemade scripts.

### hiPSC cardiac differentiation

Three hiPSC lines TROPO-GFP (mono-allelic mEGFP-Tagged TNNI1 WTC), GJA1-GFP (mono-allelic mEGFP-Tagged GAJ1 WTC), and SS109 were used in this study. TROPO-GFP and GAJ1-GFP were obtained from Coriell Cell Repositories (AICS-0037-172 and AICS-0053-016) and SS109 clone (Characterization of hiPSC cell line in Additional file 2: Fig. S1A–D, and Biomarkers of different stages of differentiation in Additional file 2: Fig. S1E, F) were generated in-house with lentivirus from skin fibroblasts of a healthy donor following the protocol of Somers et.al. [19]. For cardiac differentiation, hiPSCs were plated at 0.5 × 10^5^/cm^2^ on 12 wells Geltrex coated plates and cultivated in 1:1 medium (E8: mTeSR, 85,850, Stemcell Technologies) (Day-2). The cells were maintained in this media with daily change until reaching 100% confluence (Day 0). We followed the differentiation protocol previously published by [20]. At day 0 of differentiation, cells were washed twice with DPBS (14,040,216, Thermo Fisher Scientific) and then cultivated in Differentiation Medium (RPMI 1640 (11,875,119 Thermo Fisher Scientific), + B27 minus insulin (A1895601, Thermo Fisher Scientific), + 6-9 µM GSK-3 inhibitor CHIR 99,021 (361,571, Millipore). Media was replaced after 48 h (Day 2) with RPMI-1640 supplemented with B27 without insulin. Media was replaced after 24 h (Day 3) with RPMI-1640 supplemented with B27 without insulin and 2 µM Wnt-C59 (5148/10, Tocris Bioscience) and incubated for 48 h. At day 5, media was replaced with RPMI-1640 supplemented with B27 without insulin. From day 7, cells were cultivated with RPMI-1640 supplemented with B27 with insulin until the end of the protocol. When beating cells were observed between days 9 and 11, metabolic selection of cardiomyocytes was conducted using glucose-free RPMI 1640 (11,879,020, Thermo Fisher Scientific) supplemented with B27 and insulin for 48 h, if necessary, the medium was replenished for 2 more days.

### Transcriptomic analysis from different stages of maturation of hiPSC-derived cardiac cells

Total RNA of clone SS109 was extracted from hiPSC-CMs in different stages of maturation using TRIzol reagent followed by isolation and purification using miRNeasy (QIAGEN, USA) according to the manufacturer's instructions. RNA quality and quantity were assessed using Agilent 2100 Bioanalyzer chip (AGILENT, USA). All samples were labeled using FlashTag Biotin HSR RNA Labeling Kit (901,911, Thermo Fisher Scientific). To assess gene expression, RNA was hybridized to Clariom™ S Assay HT, human (902,969, Thermo Fisher Scientific), whereas to assess miRNA expression the biotinylated samples were hybridized to GeneChipTM miRNA 4.1 array plate (902,409, Thermo Fisher Scientific) washed and stained according to the manufacturer’s protocols. The gene and miRNA Array Plates were scanned using the GeneTitanTM Instrument (Thermo Fisher Scientific). Expression Console Software was used to create summarized expression values (CHP-files). Data were analyzed using Transcriptome Analysis Console (TAC) software generating fold-change, p-value, etc., and MATLAB homemade scripts (files are in NCBI Gene Expression Omnibus database repository record GSE188749).

### Experimental validation of gene/miRNA modulatory properties in hiPSC-CMs in vitro

hiPSC-CMs (TROPO-GFP) were transiently transfected using Lipofectamine 2000 (Thermo Fisher Scientific) diluted in OPTI-MEM (Thermo Fisher Scientific) combined with mimics following the manufacturer’s instructions at a final concentration of 30 µM of the pre-miR-124-3p, miR-512-3p, pre-miR302d-3p, pre-miR302c-3p, pre-miR-1323, pre-miR526b-5p, pre-miR30a-5p and pre-miR526b-5p. All miRNAs oligonucleotide sequences are listed in Additional file 2: Table S2.

For transfection’s negative controls we tested three different conditions: cells treated with Negative Control #1 (NTC) (mirVana miRNA Mimic-cat 4,464,059; Thermo Fisher Scientific, USA), cells only with Rb + media, and cells treated with Opti-MEM plus Lipofectamine. Among all treatments, there were no changes in EdU incorporation levels (Additional file 2: Fig. S2A), in relative levels of Calcium intracellular and Amplitude (Additional file 2: Fig. S2B), and no functional modulation of SERCA-mediated intracellular calcium levels with thapsigargin (5 μM) (Additional file 2: Fig. S2C). Based on this, Opti-MEM plus Lipofectamine (Control) were used as a negative control in all experiments.

Forty-eight hours after cell transfection, total RNA was extracted using TRIzol reagent followed by purification using a RNeasy Micro kit (Qiagen, Hilden, Germany) according to the manufacturer’s instructions. Reverse transcription was performed using SuperScript™ IV First-Strand Synthesis System (Thermo Fisher Scientific). Real-time quantitative PCR was performed using the QuantiTect SYBR® Green reagent (Qiagen, Hilden, Germany) to determine the relative gene expression of *EPM2AIP1*, *LBH*, *CASQ2*, *MICB*, *TSPAN33*, and *TMEM30B*. GAPDH was used as a housekeeping gene, and results were expressed using the 2 − ΔΔCt method. The primers used for qRT-PCR are listed in Additional file 2: Table S3.

### High-content phenotypic screening

hiPSC-CMs were plated to confluence at 1 × 10^5^ cells/cm^2^ in 96-well fluorescence plates (Wells Greiner Microplate, Black, Clear Bottom), previously treated with Geltrex (Thermo Fisher Scientific) and transfected with miRNAs on D8 or D23. Cells were fixed in 4% (vol/vol) paraformaldehyde and permeabilized with 0.5% Triton X-100/PBS for 5 min. Immunostaining was performed using anti-MYH6 (Ab15, 1:200, Abcam), anti-MYH7 (PA5-100,023, 1:200, Thermo Fisher Scientific), anti-Ki67 (ab16667, 1:200, Abcam), anti-Tom20 (sc-11415, 1:200, Santa Cruz), anti-SERCA2 a/b (ab2861, 1:200, Abcam), and anti-troponinI3 (4T21/2, 1:200, Hytest), followed by appropriate secondary antibodies conjugated with Alexa fluor 488/555/647 fluorophores (1:500, Thermo Fisher Scientific). DAPI was used to counterstain nuclei. For the high-content screening analysis, from 16 up to 64 fields of view of each well were collected using EVOS M7000 (Thermo Fisher Scientific) automated imaging system at either 20 × or 40 × magnification and analyzed using CellProfiler (4.2.0) [21]. The high-throughput analysis retrieved phenotypic information of more than 2000 single-cells per sample, for cell-based analysis, or 150–500 images for image-based analysis. For EdU staining, 5 mM 5-ethynyl-20-deoxyuridine (C10640, Thermo Fisher Scientific) was added to the cell medium for 48 h before immunofluorescence observation. Before incubation with secondary antibodies, the cells were processed using the Click-IT EdU cell proliferation kit for imaging (Thermo Fisher Scientific) according to the manufacturer’s instructions.

For Tunel staining, we used Click-iT TUNEL Alexa Fluor Imaging Assays for Microscopy & HCS (C10246, Thermo Fisher Scientific) according to the manufacturer’s instructions.

We measured mean fluorescence intensity of each protein of interest, cell area, and cell eccentricity. Violin plots and box plots, respectively, represent data from several hundreds of cells and several dozens of images acquired in at least three independent experiments, performed in triplicate.

### Immunofluorescence and confocal microscopy

TROPO-GFP hiPSC-CMs were plated to confluence at 1 × 10^5^ cells/cm^2^ in CellView Culture Slide (543,079, Greiner BioOne), previously treated with Geltrex and transfected with miRNAs on day 23. Cells were fixed in 4% (vol/vol) paraformaldehyde and permeabilized with 0.5% Triton X-100/PBS for 5 min. Immunostaining was performed using anti-Tom20 (sc-11415, 1:200), followed by appropriate secondary antibodies conjugated with Alexa fluor 555 fluorophore (1:500, Thermo Fisher Scientific). DAPI was used to counterstain nuclei. Images were acquired on an Axio Observer.Z1 (Carl Zeiss, Germany) equipped with a Confocal Spinning Disk Unit (CSU-X1; Yokogawa, Japan) and a Rolera EM-C2 EMCCD camera (Teledyne Photometrics, USA). DAPI, ssTnI, and Tom20 were, respectively, detected by sequentially illuminating samples with 405 nm, 488 nm, and 561 nm laser lines through a C-Apochromat 40x/1.20 W Korr objective (Carl Zeiss, Germany). Image analysis was performed in CellProfiler after preprocessing in Fiji [22]. Briefly, “Subtract Background” filter plugin (rolling ball radius = 5) was applied to Tom20 images in order to reduce background and highlight mitochondria. Preprocessed Tom20 images were then processed in CellProfiler in order to extract mean Tom20 intensity and distribution in segmented mitochondria. Representative images were further processed by using the auto function of “Brightness/Contrast” plugin in Fiji, in order to highlight subcellular localization of proteins of interest.

### Mitochondrial membrane potential detection by TMRM

TMRM was used for mitochondrial membrane potential detection. TROPO-GFP hiPSC-CMs were plated to confluence at 0,7 × 10^4^ cells/cm^2^ plated in 96-well plates were incubated in 25 nM TMRM (Thermo Fisher Scientific), 200 nM MitoTracker Deep Red FM (to mitochondrial localization, Thermo Fisher Scientific) and 8.1 µM Hoechst (to detect nucleus, Thermo Fisher Scientific) diluted in RB^+^, at 37 °C for 30 min in a dark environment. Thereafter, cells were washed with PBS and images were acquired at the microscopy Evos M7000 (Thermo Fisher Scientific). Fluorescence intensity was analyzed according to the manufacturer’s instructions.

### Morphology and contractile structures with MorphoScript

To characterize the hiPSC-CM morphology as well as the organization of their contractile structures, hiPSC-CM were dissociated with TrypLE Select 10x (A1217701, Thermo Fisher Scientific) for 15 min at 37 °C and replated on a 96-well plate with 10 μM ROCK inhibitor Y-27632 (Y0503, Sigma-Aldrich) added to the maintenance medium of hiPSC-CM. Low confluence was used to obtain isolated cells (0, 7 × 10^4^ cells). The cells were then fixed with 4% PFA for 15 min at room temperature, washed twice with PBS and permeabilized with 0.1% Triton X-100 for 5 min. The blocking was performed with 5% bovine serum albumin (BSA, Sigma-Aldrich) for 60 min at room temperature. Alpha-actinin2 antibody (A7811, 1:500, Sigma-Aldrich) diluted in 2% BSA was used and incubated overnight at 4 °C. After incubation with primary antibody, cells were washed twice with PBS and followed by appropriate secondary antibodies conjugated with Alexa fluor 555 fluorophore (1:500, Thermo Fisher Scientific) and DAPI (1:500) for one hour at room temperature. The images were acquired using the EVOS M7000 at × 40 magnification and analyzed using the MorphoScript software at MATLAB, as described [23]. The isolated cells were segmented by the semi-automatic or manual method. For analysis, we set the window size to 15, this way each window contains at least five contractile structures labeled with a-actinin. For the rest of the parameters, the default values were kept. This analysis allows us to describe the morphology of hiPSC-CM through the measure of the sarcomeric structures organization with striation (the order) and their alignment (the dispersion) and geometric parameters such as the cell surface area and eccentricity.

### Measurement of contractile properties with contraction wave

For measurement of contractile properties, well plates containing spontaneously beating hiPSC-derived CMs (spontaneous contractions) were incubated in a thermostatic chamber (21% O2 and 5% CO2, at 37 °C), to maintain physiologic conditions. The hiPSC-CMs were visualized using EVOS M7000, equipped with a 20 × objective. Cells were allowed to stabilize for at least 10 min prior to any recordings. Movie images of beating hiPSC-CMs were recorded with a duration of 15–20 s for each position, at 30 frames per second. Using ContractionWave [24], an open-source software for large-scale analysis of cardiomyocytes contraction, we performed the analysis of hiPSC-CMs contractility, measuring parameters related to time, speed, area, and frequency.

### Calcium handling

Cardiomyocytes seeded at a density of 30,000 cells on a 96 wells plate were loaded with the calcium sensitive dye Fluo-4 NW Calcium Assay Kit (F36206, Molecular Probes) for 45 min at 37 °C. After washing and 5 min to allow de-esterification with Tyrode’s solution containing NaCl (135 mM), KCl (5.4 mM), MgCl (1.0 mM), HEPES (10 mM), CaCl2 (1.8 mM), glucose (5.0 mM) (pH 7.4), fluorescent signals were obtained upon excitation at 488 nm and the emission fluorescence signals collected at 516 nm for 20 s EVOS M7000. After all frames were imported and processed in ImageJ Software, data were analyzed using the pClamp software (version 11.2, https://support.moleculardevices.com/) to generate the Ca^2+^ transient parameters reported in this study.

The Intracellular Ca^2+^ [Ca^2+^]_i_ was measured using Fura-2, AM (F1221; Thermo Fisher Scientific). Tyrode’s free Ca^2+^ solution (final concentration of 2 µm) was incubated for 10 min at room temperature. Next, cells were incubated for more 5 min at 37 °C and washed once with Tyrode free Ca^2+^ to exclude the contribution of trans-sarcolemma Ca^2+^ influx via voltage-gated Ca^2+^ channels. Prior to measurements, cells were incubated for 5 min to enable complete de-esterification of intracellular Fura-2. Intracellular Ca^2+^ events were recorded using a 40 × objective on an Olympus IX70 microscope fitted with an IonOptix system (IonOptix, Milton, MA) at room temperature. Samples were excited at 340 and 380 nm and the emitted fluorescence was collected at 510 nm. Ca^2+^ level was measured as the ratio of fluorescence at 340 and 380 nm (340/380 nm). Calibration was performed using 1% Triton X-100 for total fluorophore release and 20 mmol/L EGTA to chelate the free Ca^2+^ [25].

To measure calcium sensitivity, spontaneously beating hiPSC-CM were stimulated by temporally limited puffs of caffeine, a pharmacological agonist of RYR2 (10 mM; Sigma) or thapsigargin, a specific SERCA inhibitor (5 µM; Sigma) for 500 seg and we analyzed between two points 200 ~ 220 and 400 ~ 440 s.

### Membrane potential

Electrical activity was recorded using the Fluovolt voltage-sensitive dye kit (Molecular Probes, USA). Fluovolt dye was diluted in Tyrode’s solution at 37° containing PowerLoad (a solubilizing agent provided in the kit) and blebbistatin (100 μM; Sigma). Cells were incubated for 10 min at room temperature followed by 5 min at 37 °C and washed once with Tyrode's solution. Fluorescence intensities (Excitation/Emission = 480/540 nm) were measured on a multi-mode microplate reader (SpectraMax iD3; Molecular Devices, USA). To acquire the signals with a maximal time resolution, the minimal sampling interval of 10 ms was chosen for this study. All experiments were performed at the controlled temperature of 37 °C. Data were analyzed using the pClamp software (version 11.2, https://support.moleculardevices.com/).

### Statistical analyses

PCR results were expressed as mean ± standard error of the mean. Immunofluorescence was represented as boxplots with median and Min to Max range. Single-cell immunofluorescence was represented by violin plots with data median. Normality (Shapiro Wilk) was verified. One-way analysis of variance with Bonferroni post hoc test or unpaired Student’s t-test was used to compare groups as appropriate. If homogeneity of variances was not confirmed, the Welch's ANOVA test was performed. When the data were not normally distributed, the Kruskal–Wallis test followed by the Wilcoxon test was used. Except for the Pearson linear correlation analysis as previously described, all statistical analyses were performed using GraphPad Prism 8.0 (GraphPad Software Inc., CA, USA). *P-*values < 0.05 were considered significant. For *p* < 0.05 = *; *p* < 0.001 = **; *p* < 0.0005 = *** and *p* < 0.0001 = ****, except when indicated.

## Results

### Segmental differential expression (DE) analysis to select candidate transcripts for modulation of differentiation

**Fig. 1:**
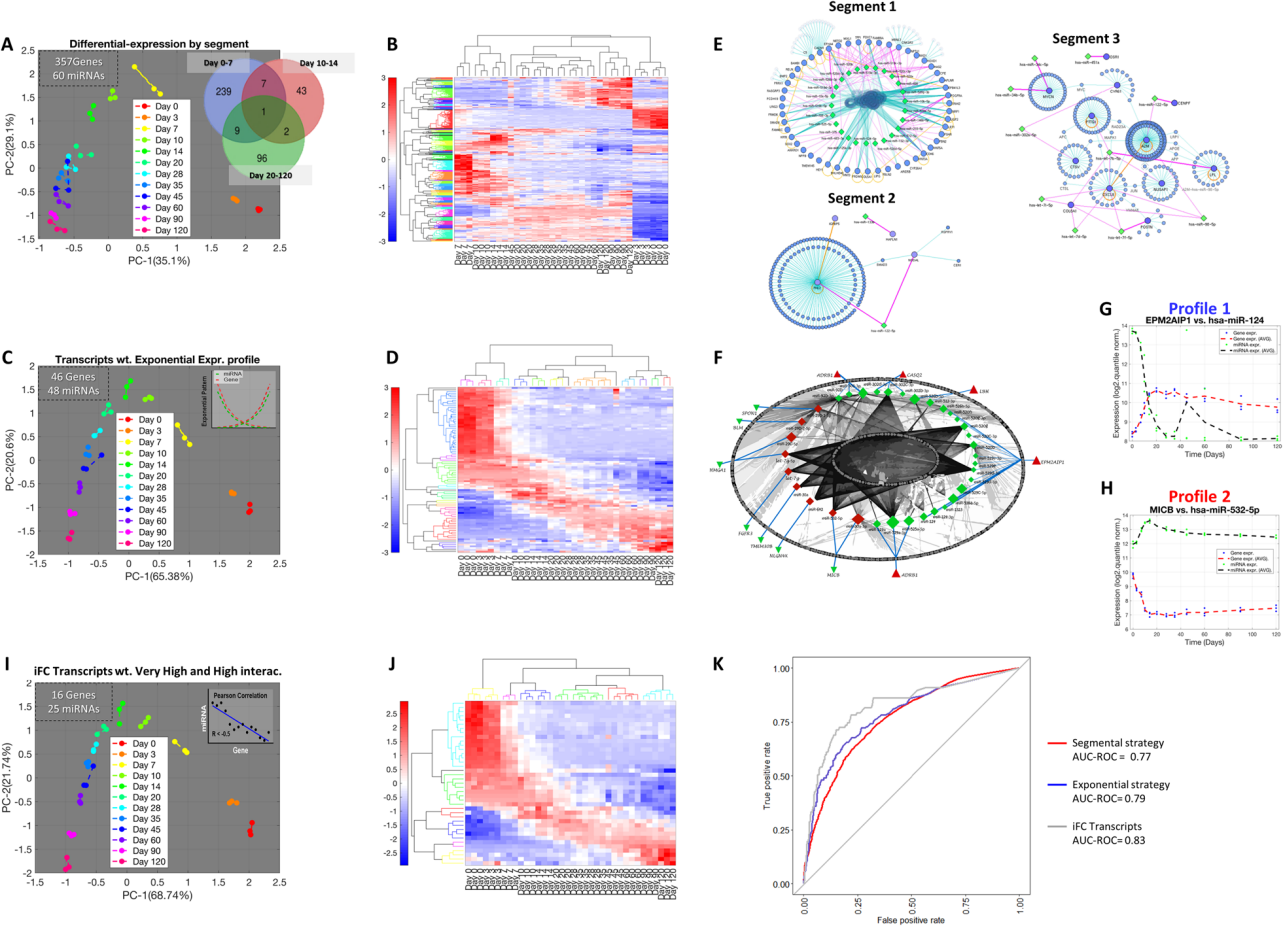
Approaches for filtering and selecting genes relevant to developing hiPSC cardiomyocytes over time. **A** PCA grouping by the segmental DE analysis strategy. Total number of DE miRNA and genes filtered by the approach located in the upper left region of the PCA. Venn diagram (right side) representing the number of DE genes per segment. **B** Hierarchical clustering analysis of the Segmental DE transcripts patterns of the in vitro model of cardiac cell differentiation. **C** PCA and **D** Hierarchical clustering of exponentially expressed transcripts from Babiarz et al. 2012 dataset. **E** DE transcripts interactions generated by I2D analysis from miRTARBase in each stage of cardiac differentiation (Segment 1: Days 0–7, Segment 2: Days 10–14, and Segment 3: Days 20–120). **F** miRNA/Gene interaction analysis using the list of exponentially expressed transcripts retrieved from I2D. Symbol sizes represent genes/miRNAs fold changes (FC) (the larger the expression, the larger the symbol and vice versa). Triangles and diamonds, respectively, represented gene and miRNA expression. Red and green symbols, respectively, denote up- and downregulated transcripts. Blue lines represent the power of the gene-miRNA interactions (the thicker the line, the stronger the interaction). **G** Representative Profile 1 (decreased miRNA expression over time and increased target gene expression) and **H** Profile 2 (increased miRNA expression over time and decreased target gene expression) graphs denoting the inverse fold-change (iFC) pattern. **I** PCA of the selected genes with iFC pattern from transcripts with high/very high interactions. Selected list of transcripts increases the Euclidian distances between samples from different groups compared to the previous lists. **J** Hierarchical clustering of iFC transcripts. **K** *ROC–AUC graphs:* Three independent random forest models were trained using the list of genes and their expression levels to predict maturation. The ROC curves display the predicted model efficiency for each group (Red line = segmental strategy, Blue line = exponential profile Strategy, Gray line = iFC (very) high interactions)

Considering the limitations to capture subtle time-dependent changes in cardiomyocytes differentiation, especially from days 20 to 120, when most of the cardiac maturation process occurs, we devised a strategy called *“*segmental DE analysis*”* to increase the resolution of the DE transcripts during this period. To characterize the small differences in transcripts associated with differentiation and maturation on hiPSC-CMs, cell samples were split into three groups representing the most significant phases of cardiac embryonic development (days 0 to 7, pluripotency-to-cardio mesoderm specification; days 10 to 14, cardiac specification; and days 20 to 120, several levels of cardiac maturation [26] (Fig. 1A, B).

Using this approach, we identified 357 DE genes and 60 miRNAs. In the first segment of cardiac differentiation (days 0–7), there were 256 DE transcripts (218 genes [190 upregulated and 28 downregulated] and 38 miRNAs [16 upregulated and 22 downregulated]). In the second segment (days 10–14), we found 53 DE transcripts (49 genes [42 upregulated and seven downregulated] and four upregulated miRNAs). In the third segment of differentiation (days 20–120), we identified 108 DE transcripts (90 genes [58 upregulated and 32 downregulated and 18 miRNAs [14 upregulated and four downregulated] (Fig. 1A). Only one gene was common for the three segments investigated (*STMN2*). Seven genes overlapped between segments 1 and 2, nine genes between segments 1 and 3, and two transcripts, one gene (*POSTN*) and one miRNA (miR-122), were common for segments 2 and 3 (Table 1).

**Table 1:** Common transcripts differentially regulated during 3 phases of cardiac differentiation in vitro

Segment overlapping	Transcripts
Days 0 to 7/days 10 to 14	APLNR, CST1, CYP26A1, HAPLN1, NODAL, NTS, TNNC1
Days 0 to 7/days 20 to 120	BHLHE40, BMP2, DLK1, EGFLAM, FREM1, LOX, NPTX1, RELN, SPON1
Days 7 to 14/days 20 to 120	POSTN, miR-122
3 segments	STMN2

Using unsupervised clustering techniques, we found that cells on days 0 and 3 were closely grouped in four sub-clusters (Fig. 1B). Accordingly, samples from day 7 grouped with a second cluster containing samples from days 10–14. Samples from days 20–35 (early-cardiomyocytes) [27–29] were grouped as also observed for cells from days 45–60. Finally, we identified a fifth sub-cluster, including samples from days 90–120 (late-cardiomyocytes) [26, 27, 27, 28, 28, 29]; Fig. 1A for principal component analysis [PCA]).

### Exponential expression analysis to improve clusterization and filtering of candidate transcripts related to differentiation

The long-term cultivation of hiPSC-CMs slightly affects their global transcriptome over time. In this scenario, specific transcripts can display more monotonic expressions, which facilitate their traceability, and may increase sensitivity for grouping the cells from clusters 0–120. To filter the list of “segmental DE transcripts” with these monotonic transcripts, we designed an algorithm to capture exponentially expressed transcripts based on a second-degree exponential fit equation (Fig. 1C).

PCA and hierarchical clustering analysis of the list of transcripts displaying exponential expression patterns improved the grouping capacity for each time point of cardiac differentiation (Fig. 1C, D). This strategy resulted in a list of 46 genes and 48 miRNAs (Additional file 1: Table S1) that fit the exponential equation (time, days, vs. expression relation). Additionally, minor differences of expression between two neighboring time points, not clear before, were uncovered by the transcripts with exponential expression shape, suggesting an improvement in the clusterization and selection of candidates associated with cardiac differentiation in vitro.

### Gene-miRNA network interactions and transcriptional profiles were identified using the exponential expression analysis

As an approach to determine whether the genes and miRNAs selected through the exponential strategy influence critical biological processes, we subjected the genes to an interaction analysis with their miRNAs targets using the miRTARBase database and the algorithm I2D + NAViGaTOR. Biological relevance was inferred when the network displayed high connectivity (interaction) among genes and miRNAs.

The mRNA transcripts found in the segmental strategy within segment 1 uncovered 124 interactions, 42 with high or very high confidence calculated using miRTARBase (Fig. 1E). In segment 2 (days 10–14), 931 interactions were identified, 112 with high or very high confidence. Finally, in segment 3 (days 20–120), only five of 23 were classified as high-confidence interactions (Additional file 1: Table S1).

Next, we used the transcripts filtered using the exponential strategy for the interaction analysis, considering the confidence class to select only high and very high-confidence gene-miRNA pairs (Additional file 1: Table S1). This filtering step resulted in 324 interactions among 29 genes and 51 miRNAs (Additional file 1: Table S1) represented in a schematic network (Fig. 1F). Interestingly, genes such as *VASH2*, *EPM2AIP1*, *NLGN4X*, *LEF1*, and *MICB* were found in more than 20 pairs of interactions. The miR-520 family (miR-520 g, h, d, c, and b, respectively) was the most prevalent, with each member displaying more than 15 pair interactions (Additional file 1: Table S1).

In Fig. 1G, H, we illustrate two representative patterns of gene-miRNA pair interactions along the different stages of the differentiation. This temporal plotting denoted the inverse fold-change (iFC) of expression between the gene and the miRNA pair, supporting the notion of a functional miRNA influence on its target gene during cardiac differentiation.

These iFC expression patterns prompted us to create a quantitative score to classify the interactions' power and then filter the transcripts according to the iFC profile.

### The use of Pearson’s correlation to select the transcriptomic signature in the cardiac differentiation model (Babiarz dataset)

We subjected gene-miRNA pairs to Pearson linear correlation coefficient analysis to obtain *R*-values and *P*-values for each transcript pairwise because we wanted to focus on the transcripts with more linear patterns of inverse expression. This analysis resulted in a list of interactions with positive or negative Pearson *R*-values and respective *P*-values. Negative Pearson *R*-values were considered validated interactions, and positive Pearson *R*-values considered non-validated interactions (Additional file 1: Table S1).

Pearson's correlation is an assessment for linear correlations; hence, more highly negative Pearson *R*-values represented gene-miRNA pairs displaying higher linearly inversed segments over time. In contrast, pairs with fewer segments in linear shape were represented by less negative Pearson *R*-values. We then considered only gene-miRNAs interactions with Pearson *R*-values < − 0.5 for further assessment. Thus, 42 of the 324 interactions (16 of the 29 genes and 25 of the 51 miRNAs) displayed high/very high confidence and significant negative Pearson correlations (Additional file 1: Table S1).

Next, we subjected these significant interactions to unsupervised PCA and hierarchical clustering assessments (Fig. 1I, J). Notably, the hierarchical clustering analysis resulted in a transcriptomic signature that uncovered mild differences in expression among the stages of cardiac differentiation in vitro (Fig. 1J).

To determine whether the set of genes filtered by the previous approaches would predict the various stages of maturation with greater precision, we trained a random forest algorithm with the three sets of genes present in each of the transcript selection steps (Fig. 1K). After training the model, we assessed the prediction efficiency among the groups using receiver operating characteristic (ROC) curves and calculated the area under those curves (AUC). For Group 1, where we selected all genes using the segmental strategy (10,512 observations), the prediction accuracy was 77%. In Group 2, for genes selected by the exponential strategy (2,376 observations), the ROC curve displayed accuracy of 79%. Finally, in Group 3, for the set of genes filtered by the iFC transcripts with high and very high interactions (1,044 observations), the accuracy was 83%, indicating that the latter strategy displayed improved prediction capacity to associate gene/miRNA pairs along the differentiation, despite the more stringent transcript pairs selection (Fig. 1K).

Collectively, these results suggest that transcripts with higher sensitivity for grouping hiPSC-CMs along the maturation time can be identified by using the proposed filtering strategy (segmental approach, transcripts with exponential expression, and negative iFC). We began with a universe of 20,543 unique transcripts (19,398 genes and 1145 miRNAs) and reached 41 transcripts (less than 0.2% of total, 16 genes, and 25 miRNAs), classified as transcripts with high or very high-confidence interaction to capture the critical interactions.

### In silico validation revealed the robustness of the hiPSC-CMs time-regulated transcripts during differentiation using different datasets

We next determined whether this molecular signature persisted in multiple hiPSC and human embryonic stem cells (hESCs) cell lines, and diverse differentiation protocol specificities in silico (Additional file 1: Table S1).

We performed an in silico validation of the iFC interactive transcripts against eleven datasets for gene expression and three human datasets for miRNA expression available in the literature (the gene and miRNA expression were simultaneously measured only in one dataset) (Additional file 1: Table S1). All datasets were obtained from the Gene Expression Omnibus. A detailed description of each study is found in Additional file 1: Table S1.

**Fig. 2:**
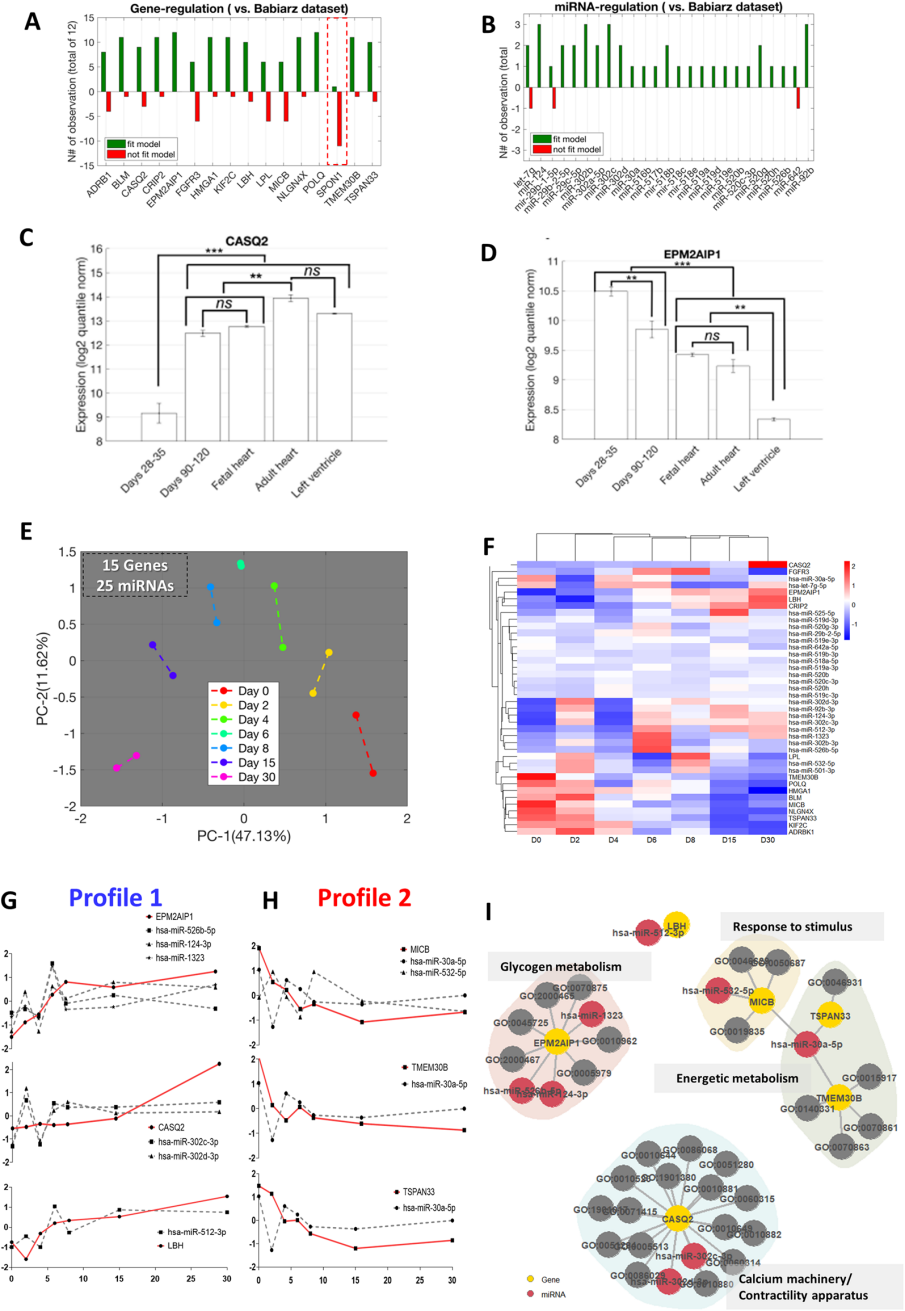
In silico protocol reveals robust gene signature among different databases and predictshiPSC-CMs differentiations stages. **A** Genes and **B** miRNAs were, respectively, subjected to a comparative analysis of their fold-change regarding the expression of the most mature sample available in each study versus the Day 0 (pluripotent stem cell samples). The side of the expression change was compared to the observed in the Babiarz et al. 2012 dataset. Positive (green bars) and negative (red bars) observations mean that fold-change was, respectively, driven to the same or opposite side observed in the model samples. *SPON1* was observed in 10 out of 12 databases, once in the same fold-change side of in vitro model and nine times on the opposite side (Additional file 1: Table S1). **C** and** D** Bar plots with gene expression levels for hiPSC-CMs at days 28 to 35, days 90 to 120, human fetal heart tissue, human bulk adult heart tissue, all n = 6, and human left ventricle tissue (*n* = 3) were subjected to statistical analysis aiming to demonstrate the potential for modulation using miRNA pairs in future experimental approaches. * *P* < 0.05, ** *P* < 0.001, *** *P* < 0.0001. A more in-depth analysis can be found in Additional file 1: Table S1. **E** The next validation step was to develop our hiPSC model experimentally and subject the filtered genes (40 interactions) with high and very high-confidence interaction to PCA. **F** Heatmap representation of the 15 genes with iFC and high and very high-confidence interaction in our experimental model (graph represented as a z-score of log2 expression to adjust original scales). **G** Graphs of temporal gene/miRNAs expression with iFC pattern in our hiPSC-CM model for Profile 1 and **H** Profile 2, please note that the significant iFC was calculated as shown in Table 2.** I** Network representing the interaction of the selected genes and miRNAS from our *in vitro* validation associated with Gene Ontology (GO) terms representing the main pathways associated with each gene. Full descriptions of GO terms can be found in Additional file 1: Table S1

Using an adaptation of the Kendall rank correlation matrix, we created a model to calculate how many of the transcripts concorded within their expression levels over time from the Babiarz dataset compared to all 15 datasets. We found that 15 of the 16 selected genes and all miRNAs displayed the expected behavior in at least 50% of the human datasets (Fig. 2A, B). The gene *SPON1* did not fit the model in more than half of the datasets and was disregarded (Fig. 2A). For this calculation of model fitting, we considered only datasets where probes/sequencing were available (Fig. 2A, maximum of 12 datasets, and Fig. 2B, maximum of 3 datasets). In cases where a particular transcript was not covered in one or more datasets, the sum presented in the chart was smaller than the maximum value of datasets. This approach resulted in 40 interactions among 15 genes and 25 miRNAs.

**Fig. 3:**
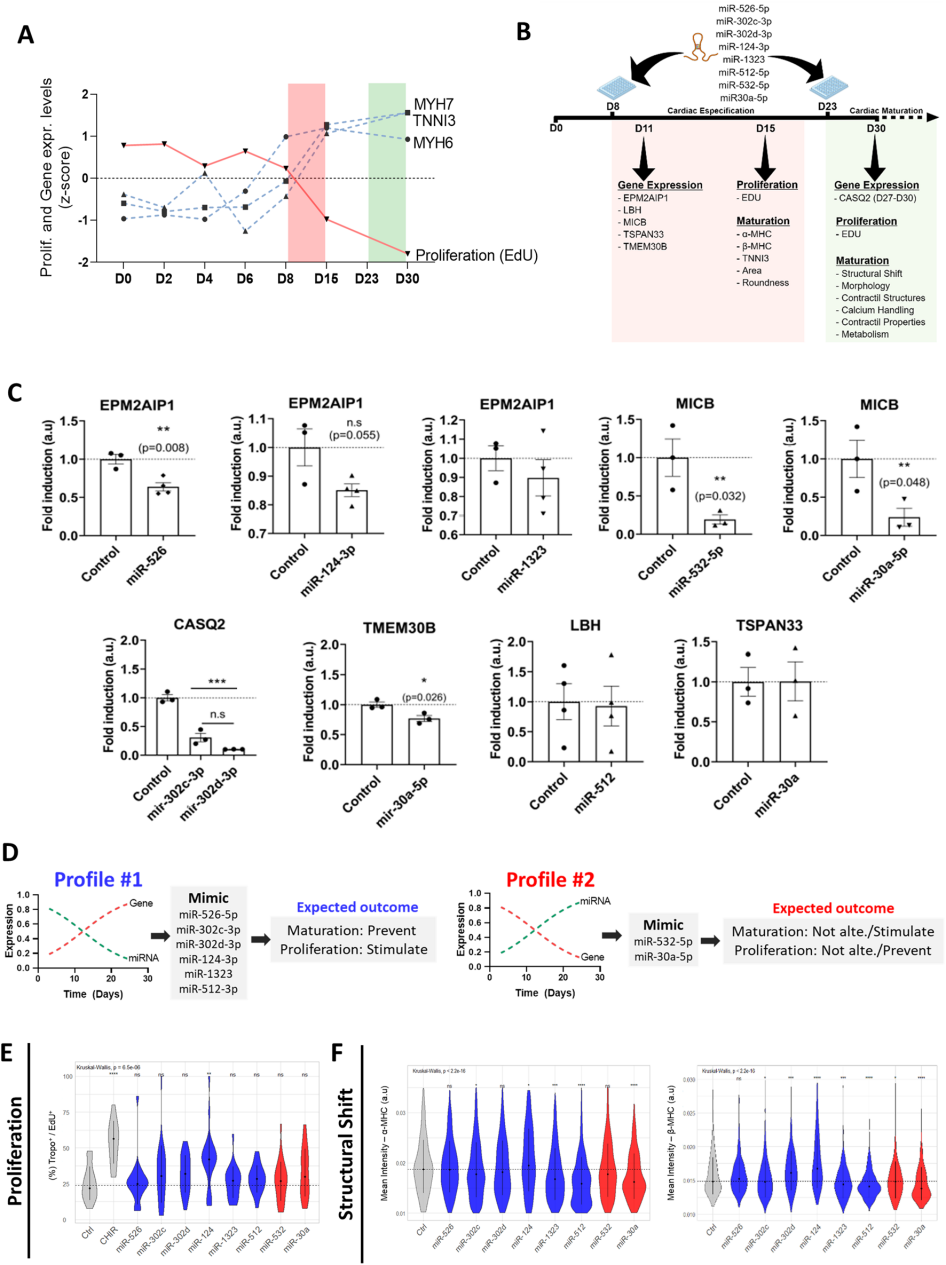
Schematics of experimental design for assessments of gene/miRNA interactions and proliferation–maturation axis of D15 on hiPSC-CMs TROPO-GFP. **A** Proliferation and maturation plot combined (EdU-positive cardiomyocytes vs. *TNNI3*, *MYH6*, and *MYH7* gene expression) of the differentiation time of hiPSC-CMs. The red box between D8-D15 and green box D23-D30 indicates the time windows used to validate miRNAs effects in the proliferation/maturation axis. **B** Schematic overview of the study. **C** Expression levels of possible gene/miRNAs interaction accessed on D11 or D30 after target-miRNAs mimics on hiPSC-CMs (*N* = 3–4 independent differentiation experiments). **D** Schematic to represent miRNAs present in Profiles 1 and 2 used in the concordance score for assessment of proliferation and maturation predictors, respectively, with expected outcome for mimic transfection given each profile characteristics. **E** hiPSC-CM proliferation estimated using the EdU assay. Results were plotted as the percentage calculated by the number of cTnI cells with EdU-positive nucleus versus all the cTnI positive nucleus. **F** Structural shift analysis based on cell mean fluorescence intensity of alpha-myosin heavy chain (*MYH6* gene) and beta-myosin heavy chain (*MYH7* gene) for each experimental group. Statistical significance treatment vs control (Ctrl) are represented as * *p* < 0.05, ** *p* < 0.001, *** *p* < 0.001. N = 3–4 independent differentiation experiments

Furthermore, from the Babiarz et al. dataset we selected two genes, *CASQ2* (Fig. 2C) and *EMP2AIP1* (Fig. 2D), that displayed iFC interactions to assess their expression levels in early (28–35) and late iPS-derived cardiomyocytes (90–120), whole fetal and adult heart and adult left ventricle samples. This analysis is consistent with the role of *CASQ2* (regulated by miR-302c-3p and miR-302d-3p) and *EPM2AIP1* (regulated by miR-124-3p) as candidates to modulate cardiac differentiation in vitro since their expression levels differed in immature cardiomyocytes (Days 28 to 35) and tissue cardiac samples (fetal or adult, Fig. 3C, D). The expression level of other selected genes (*LBH*, *TMEM30B*, and *TSPAN33*) compared with cardiac tissues are available in Additional file 1: Table S1.

Next, we performed an enrichment analysis of the candidate transcripts miRNA/pairs using Enrichr and miRSystem to uncover associations between candidate pairs and expressed genes in cardiac muscle fiber, bulk heart, ventricle, and atrium tissue samples. Gene Ontology (GO) analysis in Molecular Function revealed a calcium handling association with the transcripts (Additional file 2: Fig. S3A). In contrast, miRNAs were mainly associated with developmental biology, cell cycle regulation, calcium-related functions, and molecular functions (by GO molecular analysis), including transcriptional activity, chromatin binding, and all molecular functions associated with cardiac development dynamics (Additional file 2: Fig. S3B).

These data demonstrate that genes and miRNAs selected by the proposed filtering steps are consistently altered during cardiac differentiation. The relevant transcripts remained valid, and maintained the same expression trend regardless of differentiation protocols, laboratories, cell lineages (and species), deserving further experimental assessment.

### Experimental validation of the iFC-based strategy on the selected transcripts

To validate the expression level of genes and miRNAs selected by the proposed approach, we developed a hiPSC-CM model in our laboratory and evaluated the transcriptional levels at different intervals over 30 days of differentiation.

We used the SS109 clone generated in-house (Additional file 2: Fig. S1A–F) and sampled total RNA on days 0, 2, 4, 6, 8, 15, and 30 over the cardiac differentiation and assessed the DE of transcript/miRNA pairs using a microarray platform.

We compared changes in the gene expression profiles during the differentiation process using microarray analysis (Additional file 2: Fig. S1E). After induction with CHIR on D0, pluripotency markers, NANOG, SOX2, and OCT4, were downregulated. On the other hand, mesodermal genes, MIXL1, ROR2, and Brachyury, were transiently upregulated from D0 to D6. The cardiac mesoderm marker, MESP1, also displayed a transient expression on D2–4. PDGFR-*α*, another cardiac mesodermal progenitor cells, was significantly upregulated from D2 to D30. Transcription factors, GATA4, TBX20, ISL1, TBX5, Nkx2-5, and MEF2c, play a critical role in coordinating regulation of cardiac progenitor differentiation. Among these genes, GATA4 was first upregulated, followed by TBX20 and Isl1. TBX5, NKX2-5, and MEF2C, started to increase on D4. Expression of genes encoding cardiac contractile proteins, MYL7, MYL2, TNNI1, TNNI3, were detected at different time points, while MYL7, and TNNI1 were upregulated first on D4. HCN4 expression, which is a channel producing a pacemaker current, was upregulated around D8-D15 consistent with the beginning of spontaneous beating. Between days D8-15, genes related to non-cardiac cells CDH5, SOX17, and FOXA2, were downregulated immediately after metabolic selection.

We performed PCA and clustering to group the in silico validated transcripts (40 interactions, 15 genes, and 25 miRNAs) and their expression values (Fig. 2E, F).

We then calculated the Pearson linear correlation coefficient for all 40 interactions among 15 genes and 25 miRNAs (in silico validated signature*)* to obtain the iFC statistics. Thirty-one of 40 interactions (among 13 genes and 23 miRNAs) displayed iFCs compatible with the previous observation (Additional file 1, Additional file 2: Table S1). Sixteen interactions (among 8 genes and 12 miRNAs) displayed Pearson *R*-values < − 0.5 (Table 2).

**Table 2:** Interactive transcriptional signature validation and ranking by gene/miRNA negative correlations during cardiac differentiation in vitro

miRNA	% in− silico valid (%)	Gene	% in− silico valid (%)	*P*-value (corr.)	Pearson* R*-value
miR− 124− 3p	100	EPM2AIP1	100	0.0002	− 0.8313
miR− 512− 3p	100	LBH	83	0.0002	− 0.8292
miR− 302d− 3p	100	CASQ2	90	0.0004	− 0.8103
miR− 1323	100	EPM2AIP1	100	0.0007	− 0.7938
miR− 532− 5p	100	MICB	86	0.0008	− 0.7888
miR− 526b− 5p	100	EPM2AIP1	100	0.0027	− 0.7367
miR− 30a− 5p	100	TSPAN33	91	0.0044	− 0.7101
miR− 302c− 3p	100	CASQ2	90	0.0068	− 0.6856
miR− 519a− 3p	100	LBH	83	0.0170	− 0.6244
miR− 520 g− 3p	100	EPM2AIP1	100	0.0216	− 0.6060
miR− 519a− 3p	100	CRIP2	92	0.0227	− 0.6023
miR− 30a− 5p	100	MICB	86	0.0282	− 0.5844
miR− 520 g− 3p	100	LBH	83	0.0357	− 0.5639
miR− 525− 5p	100	CRIP2	92	0.0558	− 0.5216
let− 7 g− 5p	67	FGFR3	50	0.0578	− 0.5179
miR− 30a− 5p	100	TMEM30B	92	0.0803	− 0.5029

Following, we selected the high-confidence interaction (Pearson’s R < − 0.5 and *P*-value < 0.01) pairs to depict their expression profiles over time (Fig. 2G, H). Three genes and 6 miRNAs fit into Profile 1, displaying a progressive increase in gene expression levels accompanied by a reduction in miRNAs levels over time (Fig. 2G). In contrast, 3 genes and 2 miRNAs displayed the inverse Profile 2, where a progressive reduction in gene expression accompanied an increase in miRNAs levels (Fig. 2H).

By using the high-confidence interaction pairs, we performed a Gene Ontology (GO) analysis, and plotted the results in a network, representing the miRNA-Gene-Pathway relationship, which revealed gene-specific associations to different clustering pathways (Fig. 2I). The *CASQ2* gene associated mainly with calcium machinery and contractility apparatus pathways, whereas *EPM2AIP1* gene correlated with pathways in glycogen metabolism. The miR-30a-5p which connected three different genes demonstrated that, *TSPAN33* and *TMEM30B* were linked with energetic metabolism pathways, and the gene MICB to cell response to stimulus. For the gene LBH, no ontology term was associated with statistical significance (*p*-value < 0.01 and p-adj. < 0.05). Of note, 5 of 8 miRNAs correlated with proliferation pathways, including Hippo and Wnt (Additional file 2: Fig. S3C).

These findings are consistent with the assumption that high iFC transcripts scores influence cardiac proliferation and differentiation.

### miRNAs selected by the iFC strategy modulate expression levels of their target genes

We next tested whether the selected miRNAs modulate their targeted genes using mimic miRNAs. We chose the first eight miRNAs shown in Table 2 and all genes showing interaction and displaying significant iFC (Pearson’s *R* < − 0.05, *p*-value < 0.01) by day 8 of the cardiac differentiation (6 genes and 8 miRNAs), to be tested.

For this evaluation and subsequent screenings, we focused into two differentiation stages, the transition from the early mesodermal proliferative phase and the cardiomyocytes differentiation stage. The second, is the period where the hiPSC-CMs are considered “mature” and present robust phenotypes associated with cardiomyocytes. The early time window corresponds to the transition between the activation of specific maturation protein markers represented by their genes *MYH6*, *MYH7*, and *TNNI3* and the reduction of proliferation rates while in the latter interval these events have reached a new steady state (Fig. 3A). We assessed the mimetic-based assays (depicted in Fig. 3B) in the transition period at day 8 of differentiation that marks the beginning of maturation [26, 30–32] since the later differentiation interval (day 15 to 30) the changes are less pronounced.

The effectiveness of reducing the target expression at day 11 (72 h’ post-transfection) was tested by transfecting the hiPSC-CMs (TROPO-GFP clone) on day 8 with each mimetic molecule, except for *CASQ2* that the transfection was performed on day 27, because the inverse expression of the transcript/miRNA pair occurred later (Fig. 3C). Using this approach, we validated the decreased expression in 5 of 9 interactions (Fig. 3C). In fact, the expression of *EPM2AIP1* was downregulated by miR-526b-5p (*p* = 0.008 vs. Ctrl hiPSC-CMs), displayed a trend for miR-124-3p (*p* = 0.055) and was not affected by miR-1323 exposure. *MICB* expression was downregulated by miR-532-5p (*p* = 0.032) and miR-30a-5p (*p* = 0.048). *CASQ2* expression was downregulated by miRs 302c-3p (*p* = 0.00026 vs. Ctrl) and 302d-3p (*p* = 0.00006), and *TMEM30B* expression was downregulated by miRNA-30a-5p (*p* = 0.026). In contrast, limb-bud and heart (*LBH*) and *TSPAN33* were not affected by the mimics’ exposure.

### Candidate miRNAs affect traits associated with maturation phenotypes on responsive early hiPSC-CMs (D15)

We then evaluated the role of the miRNAs on cellular characteristics during the differentiation of hiPSC-CMs (D8-D15) using the TROPO-GFP hiPSC-CM (Efficiency of cardiomyocytes differentiation is shown in Additional file 2: Fig. S4A, B) and the SS109 clones.

We observed that six of 8 miRNAs displayed the Profile 1, characterized by decreased miRNA expression and increased target gene expression over time (Fig. 3D). We hypothesized that delivering the miRNA mimetic would downregulate the targeted genes and sustain or increase the proliferative state of the cells, delaying or preventing the cardiomyocytes differentiation (maturation characteristics). In contrast, two miRNAs displayed Profile 2, characterized by increased miRNA expression accompanied by downregulation of target gene expression (Fig. 3D). We hypothesized that delivering the mimetic miRNAs would further stimulate cardiomyocytes differentiation characteristics (or maturation may remain unchanged due to saturation and lack of response to additional miRNA).

We used a high-throughput imaging system for single-cell analysis to assess the miRNAs mimics effects on hiPSC-CM proliferation (EdU-positive cells) and structural and morphological characteristics associated with differentiation on hiPSC-CMs at day 15 (Fig. 3 E, F).

As indicated before, EdU incorporation in cardiac troponin positive cells displayed a reduction in hiPSC-CMs proliferation rate over time (Fig. 3A)*.* We used 2 µM of CHIR99021 exposure as a positive control for increased proliferation on day 8 of differentiation of hiPSC-CMs (Fig. 3E) [33] and observed that only miR-124-3p increases the rate of hiPSC-CMs proliferation compared to control (*p* = 0.019) while all the other groups remained unchanged after the exposure to the seven miRs (Fig. 3E).

For the maturation-associated characteristics of hiPSC-CMs, we used the cardiac-specific markers α-MHC (gene *MYH6*) and β-MHC (gene *MYH7*), as cardiomyocytes structural switch is associated with maturation [34, 35]. The transcriptional levels of these two proteins increased over time during cell maturation, as observed in our temporal analysis of hiPSC-CM differentiation (Fig. 3A). We observed that miR-124-3p increased *α*-MHC and β-MHC protein levels, miR-302d-3p increased only *β*-MHC, and mir-302c-3p, miR-1323, miR-512-3p and miR-30a-3p decreased both *α*-MHC and *β*-MHC. Thus, 5 of the 8 miRNAs increased the *β*-MHC/*α*-MHC ratio, namely, miR-302d, miR-124, miR-1323, miR-512, and miR-30a (Additional file 2: Fig. S4C).

We also observed decreased cardiac troponin I (cTNI) levels for all treatments, suggesting a reduction of cell maturation (Additional file 2: Fig. S4D). All miRNA mimics increased cell area compared with controls, whereas cell eccentricity (i.e., roundness) remained unchanged (Additional file 2: Fig. S4E).

Functional parameters such as ion channel changes and the switch to fatty acid metabolism are also associated with cardiomyocytes maturation, but the expression level of these gene markers remained unchanged after treatments with miRNAs at the D15 (Additional file 2: Fig. S4F, G).

Considering the heterogeneity of hiPSC-CMs and the cell’s plasticity at D15 stage, these results indicated an overall decrease in cTnI and the sarcomeric isoform switching from *α*-MHC to *β*-MHC. The inconsistent pattern of actions among the miRNAS in modifying sarcomeric proteins may reflect the immaturity of the cells at this stage and reflect the ability of all miRNAs to influence hiPSC-CMs structural features during differentiation.

### The candidate miRNAs modulate the proliferation and maturation characteristics of hiPSC-CMs at D30 of differentiation

We then analyzed the microRNAs’ effects on cell traits associated with more mature cardiomyocytes using the TROPO-GFP hiPSC-CM, GAJ1-GFP, and the SS109 clones.

The cells were transfected on day 23 of differentiation and the observations performed 7 days later, on day 30 of differentiation.

**Fig. 4:**
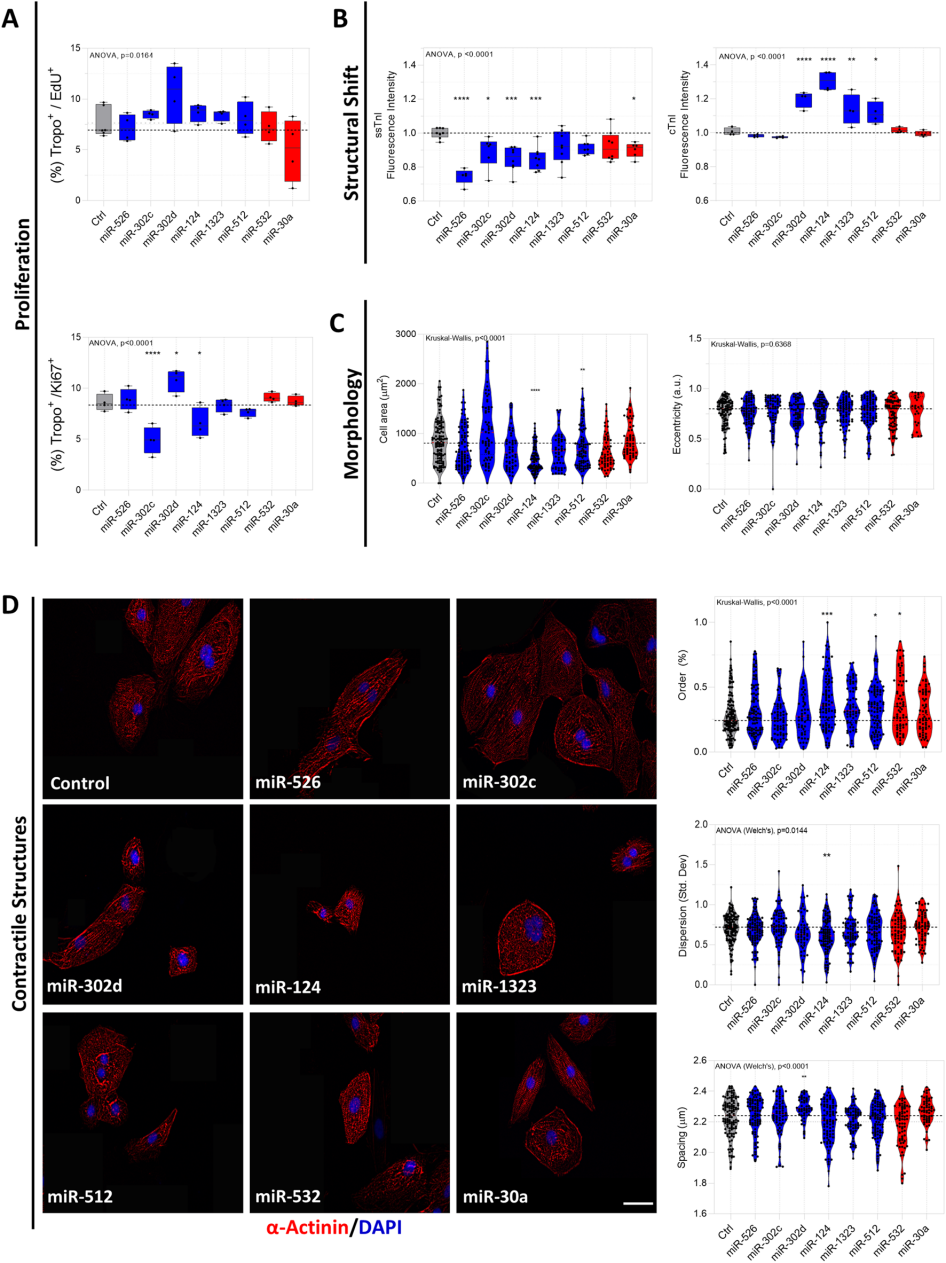
iFC-based interactive transcripts modulate the proliferation–maturation axis of hiPSC-CMs TROPO-GFP in vitro at D30. **A** hiPSC-CM proliferation estimated using the EdU assay and immunofluorescence-based detection of Ki67^+^. Results were plotted as the percentage calculated by the number of cTnI cells with EdU/Ki67 positive nucleus versus all the cTnI positive nucleus* n* = 4 independent differentiation experiments. **B** Structural shift analysis based on image mean fluorescence intensity of ssTnI (*TNNI* gene) and cTnI (*TNNI3*) for each experimental group* n* = 4–6 independent differentiation experiments. **C** Cell morphology analysis based on area and eccentricity measurements. **D** Representative images of cells treated with selected miRNAs and subjected to immunofluorescence for *α*-Actinin (Scale bar = 40 μm). Order, dispersion, and spacing of α-Actinin filaments were calculated on MorphoScript, a MATLAB-based image analysis toolbox (*N* = 91–142 cells per generated from two independent transient transfections originating from the same differentiation). Dashed lines represent the median of control cells in each graph. Statistical significance is represented as * *p* < 0.05, ** *p* < 0.01, *** *p* < 0.001 and **** *p* < 0.0001

MiR-302c-3p and miR-124-3p decreased the proportion of positive cells for cTnI^+^ and Ki67^+^ in TROPO-GFP hiPSC-CMs, whereas miR-302d-3p displayed the opposite effect (Fig. 4A). In the second cell line, SS109, miR-526b-5p, miR-302c-3p, and miR-124-3p also decreased the proportion of Ki67 cells (Additional file 2: Fig. S5A). For the EdU analysis, we found that the miRNAs treatment resulted in no changes in the proportion of positive cells for cTnI and EdU using the TROPO-GFP hiPSC-CMs (Fig. 4A).

We also assessed a maturation indicator at D30, by examining the shift from slow skeletal troponin I (ssTnI; *TNNI1*) to cardiac troponin I (cTnI; *TNNI3*) [36, 37] and observed that miR-302d-3p and miR-124-3p induced a transition from ssTnI to cTnI, corroborating the findings observed at D15. Three of the miRNAs (miR-526b-5p, miR-302c-3p, and miR-30a-5p) reduced ssTnI expression without a correspondent increase in cTnI, whereas miR-1323 and miR-512-3p increased cTnI without reducing ssTnI levels (Fig. 4B).

We also assessed morphology and contractile structures associated with maturation using MorphoScript [23]. The image analysis revealed that miR-124-3p and miR-1323 overexpression decreased cell area, whereas the eccentricity of cells exposed to miRNAs remained unchanged (Fig. 4C). On the second clone, the SS109, only miR-302d-3p decreased area while the remaining miRNAs did not modulate cell’s area and eccentricity (Additional file 2: Fig. S5B).

Sarcomeric organization and alignment are associated with cell maturation, therefore, we analyzed hiPSC-CM structure organization. We observed that miR-124-3p, miR-512-3p, and miR-532 increased the order, indicating these miRNAs increase the number of organized actinin areas compared to control cells. However, only miR-124-3p decreased the dispersion of striation. In addition, we analyzed the spacing between sarcomeres and observed that only miR-302d-3p increased this parameter (Fig. 4D). These results indicated that miR-124-3p enhances the actinin organization of hiPSC-CM, by increasing the sarcomeric order and decreasing dispersion, showing a more organized sarcomere structure compared to control cells, consistent with increased differentiation characteristics. Additionally, miR-512-3p and miR-302d-3p increased different organization features, suggesting a complex regulation of those microRNAs in the organization of the sarcomeric structures.

Altogether, these observations suggest that miRNAs associated with Profile 1 have small influence on hiPSC-CMs structural changes at later differentiation stage (D30). In contrast, miRs 302d, 124-3p, and 512-3p, modified several hiPSC-CMs differentiation features, except for the cell eccentricity. For Profile 2’s miRNAs, we found no consistent pattern of influence on the analyzed differentiation features.

### The candidate miRNAs modulate functional and metabolic maturation characteristics of D30 differentiated hiPSC-CMs

**Fig. 5:**
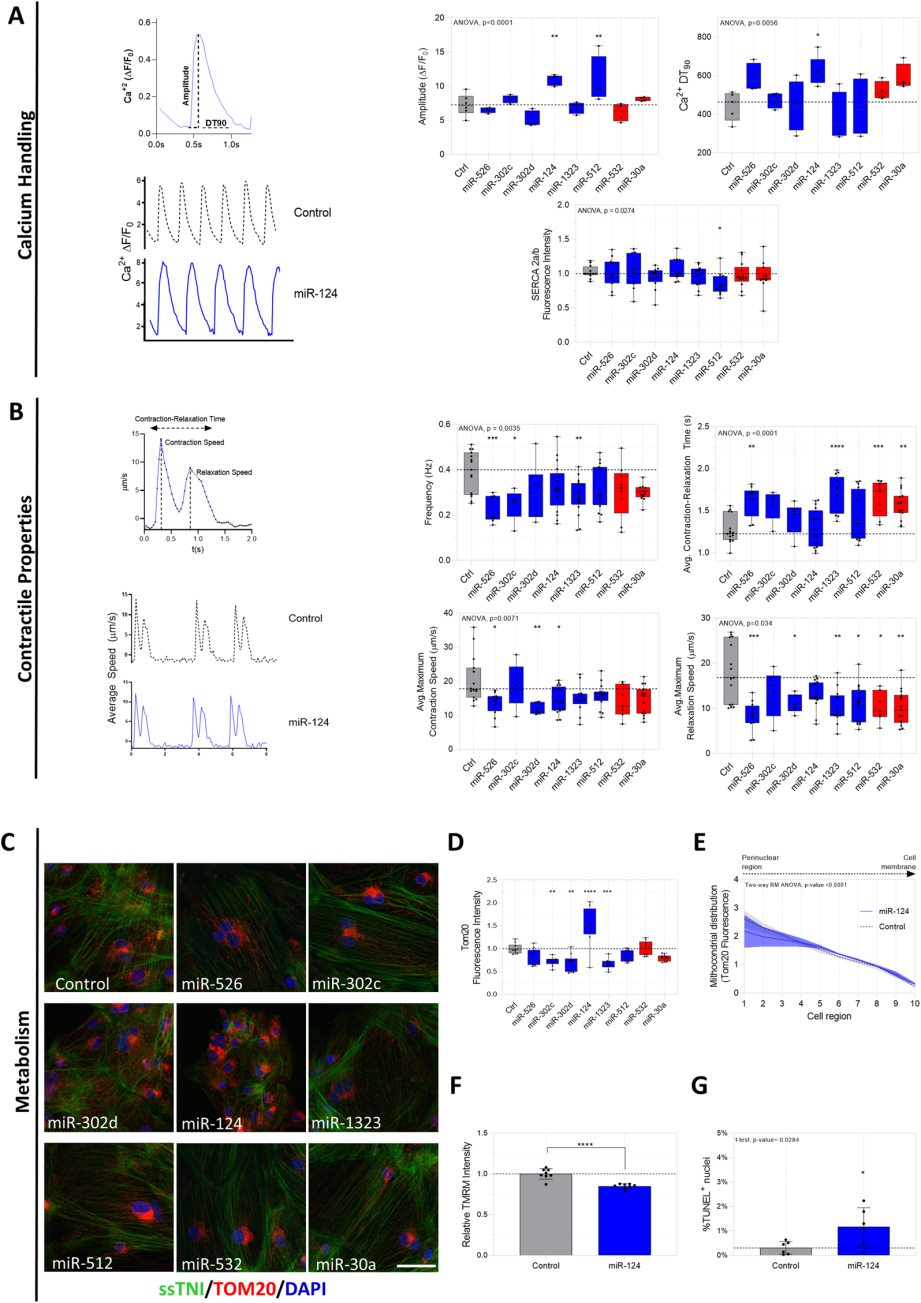
Selected miRNAs differentially regulate calcium handling, contractile properties, and metabolism markers of hiPSC-CMs TROPO-GFP at D30. **A **Top left: Schematic presenting parameters used to describe the Ca^2+^ transient, including amplitude (deltaF/F_0_) and Ca^2+^ DT_90_ (time to 90% Ca^2+^ decay, ms). Bottom left: Representative Ca^2+^ transients of control (dashed black line) and miR-124-treadted cells (solid blue line). Top right: Box plot of amplitude and Ca^2+^ DT_90_ (*N* = 4–6 independent experiments). Bottom right: Box plot of SERCA2 a/b mean fluorescence intensity (*N* = 4 independent differentiation experiments). B Top left: Schematic contraction–relaxation curve of beating hiPSC-CMs highlighting contractile parameters evaluated in this study. Bottom left: Representative contraction–relaxation curves of control (dashed black line) and miR-124-treated cells (solid blue line). Box plots of frequency, average contraction–relaxation time (Top right), average contraction speed, and maximum relaxation speed (Bottom right). Dashed lines represent the median of control cells in each graph (*N* = 4–6 independent differentiation experiments). **C** Representative confocal micrographs of control and miRNA-treated cells stained for ssTnI and Tom20 by immunofluorescence and **D** quantitative analysis of Tom20 mean fluorescence intensity (*N* = 91–142 cells per treatment from one differentiation experiment in two independent transient transfections). **E** Mitochondrial distribution (Tom20 Fluorescence) groups Ctrl e miR-124-3p-treated cells (*N* = 4 independent differentiation experiments). **F** Relative TMRM Intensity and (**G**) % of cell TUNEL.^+^. Dashed lines represent the median of control cells in each graph. Statistical significance treatment vs control (Ctrl) are represented as * *p* < 0.05, ** *p* < 0.001, *** *p* < 0.001

Considering the crucial role of Ca^2+^ transient as a functional trait of hiPSC-CM development and maturation, as well as its role for cardiomyocytes contraction and relaxation, we measured fluo-4 fluorescence under the different miRNAs treatments (Fig. 5A). MiRs 124-3p and 512-3p exposure increased the amplitude while miR-124-3p also increased the 90% decay time (DT90). MiR-512-3p and miR-302c-3p decreased fluorescence intensity of SERCA 2 a/b in TROPO-GFP and SS109 clones, respectively (Fig. 5A and Additional file 2: Fig. S5C).

Contractility properties were measured by time-lapse image recording under bright-field microscopy and the graphs illustrate the contractility parameters evaluated (contraction–relaxation time, frequency, and maximum speeds) (Fig. 5B). We observed that miRs 526b-5p, 302c-3p, and 1323 reduce frequency, contrary to expected for Profile 1 assumptions. Exposure to miRs 526b-5p, 1323, 532-3p and 30a-5p increased the contraction–relaxation time, whereas hiPSC-CM transfection with miRs 526b-5p, 302d-3p, and 124 reduced the maximum rate of contraction, whereas miRs 526b-5p, 302d-5p, 1323, 512-3p, 532-5p and 30a-5p reduced the maximum rates of relaxation (Fig. 5B).

Next, we examined the number of mitochondria marker using Tom20 fluorescence levels as proxy for development/maturation of the cells since metabolic changes are expected during the hiPSC-CM differentiation. Tom20 expression decreased by miRs 302c-3p, 302d-3p, and 1323, and increased after miR-124-3p exposure (Fig. 5C, D). On the second clone SS109, Tom20 expression decreased in response to miR-526b-5p and increased after exposure to miR-302d-3p (Additional file 2: Fig. S5D). As miR-124-3p was the only one to increase Tom20 levels as opposed to what was hypothesized in Profile 1, we investigated additional mitochondrial features such as distribution, membrane potential, and apoptosis. Mitochondrial distribution was significant only in related to the content of Tom20 in each cell region and between treatments and there was no difference between the differentiations (Fig. 5E and representative images in Additional file 2: Fig. S4H). MiR-124-3p treatment reduced the mitochondrial membrane potential (Fig. 5F). Although we observed only 2% of the Tunel-labeled cells, this increase was not pronounced suggesting that in the model of hiPSC-CM miR-124 is not associated with significant apoptosis (Fig. 5G).

These results indicate that: (1) The majority of Profile 1 miRNAs influenced functional and metabolic features, while Profile 2 miRNAs did not alter most of the investigated maturation traits. (2) miR-124-3p consistently altered the calcium transient parameters and contractility properties and mitochondrial dynamics. (3) The majority of miRNAs influenced morphological rather than functional traits.

### MiR-124-3p and miR-512-3p differentially regulate the dynamics of SR-mediated Ca.^2+^ release and reuptake and the action potential plateau phase 2 on hiPSC-CMs at D30

MiRs 124 and 512 affected the Ca^2+^ transient on hiPSC-CMs cells. Considering the essential role of the Ca^2+^ handling on hiPSC-CMs development/maturation, we investigated the miRNAs effects on intracellular calcium levels and on the main proteins associated with sarcoplasmic reticulum Ca^2+^ reuptake (SERCA) and release (RYR2), alongside the electrophysiological characteristics on spontaneously beating hiPSC-CMs.

**Fig. 6:**
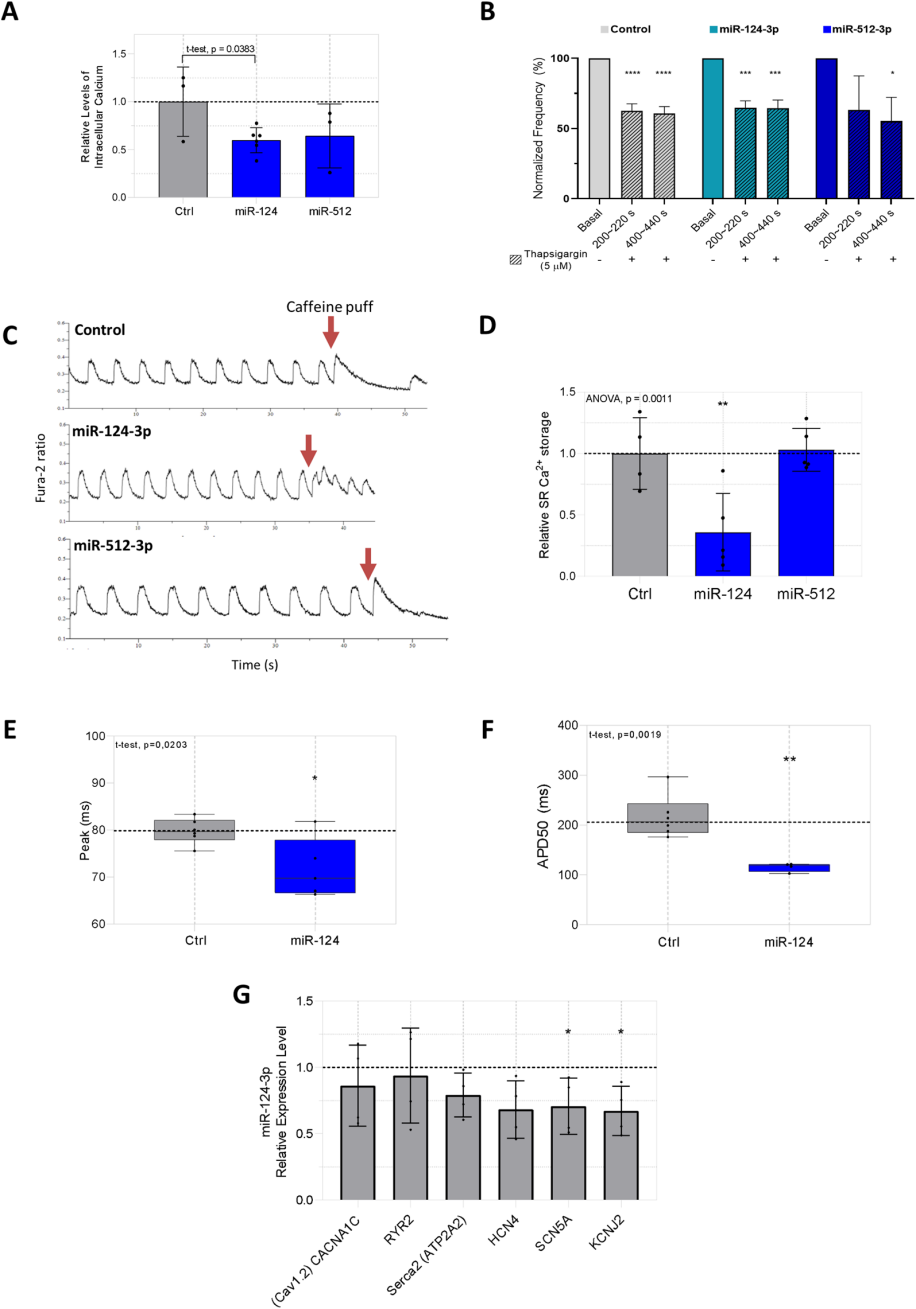
MiR-124-3p and miR-512-3p regulate the dynamics of SR-mediated Ca^2+^ release and reuptake, and the action potential on hiPSC-CMs GJA1-GFP at D30. **A** Relative Levels of Intracellular Calcium in Ctrl, miR-124-3p-treated cells and miR-512-3p-treated cells. **B** Effects of SERCA inhibitor thapsigargin (5 µm) on [Ca^2+^]I in three-point: Basal (20 s), 200–220 s, and 400 ~ 440 s in spontaneously beating cells. Normalized frequency (%) (displayed as percentage decreased versus baseline condition) (*N* = 4 independent differentiation experiments). **C** Representative Ca^2+^ transients in spontaneously beating Ctrl, miR-124-3p and miR-512-3p before and after the addition of caffeine (10 mM) (*N* = 4 independent differentiation experiments). **D** Relative levels of Ca^2+^ storage after caffeine addition were normalized to those from the same cell under the basal condition (*N* = 4 independent differentiation experiments). **E** Box plot of Peak amplitude (s) in Ctrl and miR-124-3p-treated cells. **F** Box plot of decay time 50% (ms) in Ctrl and miR-124-3p-treated cells (*N* = 5 independent differentiation experiments). **G** Expression levels of gene in Ctrl versus miR-124-3p-treated cells (*N* = 4 independent differentiation experiments). Statistical significance treatment vs control (Ctrl) are represented as * *p* < 0.05, ** *p* < 0.001

We measured the relative intracellular Ca^2+^ concentration ([Ca^2+^]_i_) using Fura-2-AM transient traces. MiR-124-3p exposure reduced [Ca^2+^]_i_ (p = 0.0383) while miR-512 displayed no significant change (Fig. 6A). SERCA functionality in hiPSC-CMs was assessed before and after exposure to thapsigargin (5 µM), an inhibitor of SERCA activity. Thapsigargin resulted in a decreased normalized frequency in control and miR-124-3p treated cells, whereas thapsigargin reduced Ca^2+^ transients only during the measurements of 400–500 s for miR-512-3p (Fig. 6B). Thapsigargin treatment for 500 s was not sufficient to block all Ca^2+^ transients in D30 differentiated cells. Relative SR Ca^2+^ release mediated by RYR2 was measured using Fura-2AM after brief exposure to caffeine (10 mM), a pharmacological agonist of ryanodine receptor (Fig. 6C). RYR2-mediated calcium release reduced in miR-124 exposure cells (*p* = 0,0032), whereas miR-512 mimic treatment did not alter SR Ca^2+^ release (Fig. 6D).

We assessed Cardiac Action Potential using a voltage-sensitive dye, Fluovolt. In miR-124-3p treated cells, we observed reduced time to peak (*p* = 0.0203) (Fig. 6E) and action potential duration 50% (APD50) (*p* = 0.019) (Fig. 6F), consistent with the expected response for a profile 1 microRNA.

We examined the expression of genes associated with the maturation process and known to control the spontaneous beating of hiPSC-CMs. Both the rapid sodium channel (*SCN5A*) and the inward-rectifier potassium channel (*KCNJ2*) were reduced in miR-124 treatments while the hyperpolarization-activated cyclic nucleotide channel (*HCN4*) remained unchanged (Fig. 6G).

In summary, we observed that miR-512 modulates calcium transients accompanied by reduction of SERCA protein function while miR-124 regulates the activity of RYR2. In addition, miR-124-3p influences the action potential, and several genes controlling the plateau phase (I_CaL_), consistent with a modulatory role of miR-124 in the control of calcium stores.

### Proposed model for the influence of the candidate miRNAs on hiPSC-CMs maturation and miR-124-4p model of action

**Fig. 7:**
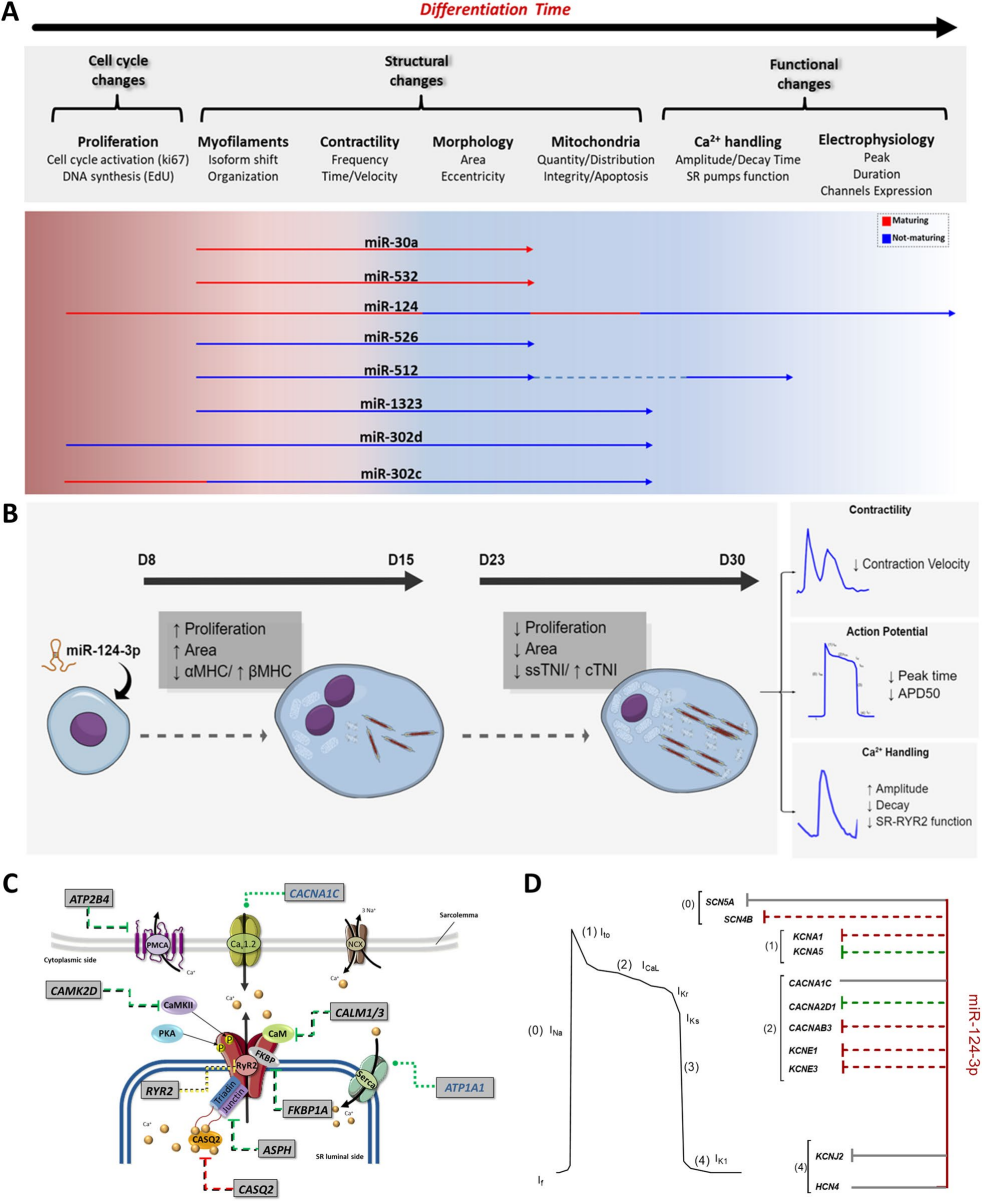
Abstract figure and proposed mechanism of action of the selected miRNAs influencing the hiPSC-CM proliferation–maturation axis. Representative model of action associated with all miRNAs analyzed **A** and miR-124 **B** regarding different maturation features analyzed that occur over time. **C** Schematic of miR-124-3p action in modulating Calcium transient by targeting genes associated with plasma membrane calcium channels, sarcoplasmic reticulum (SR) channels, and Ca^2+^ buffering-associated proteins. In the figure, green rounded arrows indicate that we accessed gene expression and found no alterations. Flat-tipped and green arrows indicate possible interactions of miR-124-3p with predicted and validated genes. Flat-tipped and yellow arrows indicate that RYR2 protein function was reduced after miR-124-3p treatments. **D** Scheme of miR-124-3p action on genes regulating different phases of the action potential. Red colors indicate that the interaction with the gene is predicted, while green colors indicate validated interactions. The graphical abstract was created with Mind the Graph

We demonstrated representative model of action associated with all miRNAs analyzed and the altered cell cycle, structural and functional features observed (Fig. 7A). Interestingly, the structural traits that change early in differentiation were the most affected by miRNAs, whereas few miRNAs influenced later functional phenotypes such as calcium handling and electrophysiology. Moreover, the profile 2 miRNAs 30a-5p and 532-5p displayed the smaller influence on the analyzed phenotypic traits. MiR-124-3p (Fig. 7B) uniquely modulated several phases of the hiPSC-CMs differentiation process examined.

We used bioinformatics to gain insight on the overall miR-124-3p target genes and their influence on hiPSC-CM calcium handling and action potential (Fig. 7C, D and Table 3).

**Table 3:** Validated targets of hsa-miR-124-3p associated with cardiac development and maturation aspects

Gene ontology term	Gene symbol	Maturation aspects	Symbol	Channels	Symbol
Atrial cardiac muscle cell development	*FHL2*	Cardiac specification	*MEF2C*	Sodium channels	SCN3B
Atrioventricular node cell development	*MAML1*		*MESP1*		*SCN9A*
	*BMPR1A*		*MIXL1*	Calcium channels	*ATP2A1*
Cardiac muscle cell development	*MTOR*	Adult cardiac related	*MYL7*		*ATP1A3*
	*SGCB*		*TNNI3*		*CACNA2D1*
	*ZMPSTE24*		*TNNC1*		*CALM1*
	*VEGFA*		*NPPB*		*CLCN2*
					*RYR2*
	*ASB2*	Cardiac subtype fate	*NR2F2*		
	*CXADR*		*SLN*		*SNTA1*
	*HNRNPU*		*GJA1*	Potassium channels	*KCNA5*
	*PLEC*		*PIAS3*		*KCNC4*
Cell growth involved in cardiac muscle cell development	*PDLIM5*				
	*AKAP13*	Cell connection	*DSG2*		*KCNE3*
Sinoatrial node cell development	*TBX3*				*KCNE4*
	*BVES*	T-tubule formation	*BIN1*		
					*KCNK1*
Ventricular cardiac muscle cell development	*LMNA*				
					*KCNK5*
	*ATG5*	Metabolism	*TFAM*		
				RYR2 modulatory proteins	CAMK2D
			*ITPR3*		*CALM1*
	*FHL2*				
					*CALM3*
					*FKBP1A*
			*ANK2*		*ASPH*
	*CDK1*				
					*CASQ2*
					CAMK2D

We identified several candidate genes targeted by miR-124-3p associated with calcium transients (Fig. 7C) and action potential (Fig. 7D), highlighting how miRNA-124-3p may affect each gene. The set of calcium transient-related genes included plasma membrane-associated calcium channels, such as *ATP2B4* and *CACNA1C*; the complex of calcium channel that mediates SR Ca2^+^ release, *RYR2*, and *FKBP1A*; and calcium buffering proteins and RYR2-associated proteins, such as *CALM1*, *CALM3*, *CAMK2D*, *ASPH*, *ATP1A1*, and *CASQ2*. Action potential-targeted genes included those related to phase 0 of rapid depolarization, *SCN4B*; phase 1 of initial repolarization (I_to_), *KCNA1* and *KCNA5*; plateau phase (I_CaL_), calcium (*CACNA2D1*, *CACNAB3*) and potassium (*KCNE1* and *KCNE3*) channels; and phase 4 of resting potential, *KCNJ2* channel gene.

## Discussion

We wanted to identify critical time-regulated transcripts to gain additional insight into the hiPSC-CM differentiation process focusing on differentially expressed gene/miRNA pairs with exponential and inverse expression along the differentiation. Using this approach, complemented by a thoroughly in silico validation with 14 datasets and an in vitro validation with alternative cell lines, we reduced the universe of more than 20,000 transcripts to 16 significant interactions among eight genes and 12 miRNAs candidates to influence hiPSC-CM differentiation via modulation of the proliferation–maturation axis in vitro. We then provided evidence that (1) this molecular signature improves tracking cardiomyocyte differentiation over time, grouping cells with improved resolution compared to currently used well-known cardiac-related genes, and (2) that these miRNAs indeed modulate proliferation, structural and functional features associated with hiPSC-CMs maturation.

Two aspects of the proposed analysis deserve attention: (1) we used an alternative method to filter candidate transcripts to discriminate minor molecular differences between groups of cells over time. The power of stratification of our strategy was significantly better within the clusters of maturation than most of the used strategies to capture differentially expressed transcripts; (2) the inverse behavior between the expression of transcripts/miRNA pairs using a negative high Pearson R-score (> − 0.5) guided the identification of transcripts that display similar regulatory behavior across various protocols using public datasets of cardiac differentiation.

The multifactorial nature of the differentiation and maturation processes required the investigation of readouts associated with cell proliferation, isoform switch to cardiac troponins, morphological parameters, sarcomeric organization, calcium handling, ions transporters, contractility functions, and metabolic markers.

Proliferation is a typical characteristic associated with immaturity, whereas mature cardiomyocytes are largely quiescent. The miRNA-124-3p stimulated proliferation on D15, but displayed the opposite effect on D30. We speculate that this finding may reflect the permissiveness of hiPSC-CMs to re-entry the cell cycle at an early phase of the differentiation process, and the role of miR-124-3p within cell cycle pathways [38, 39]. Similar actions for miR-124-3p have been reported for neuronal stem cell proliferation and differentiation through the inactivation of the Notch pathway [40]. The miR-302c-3p reduced the number of Ki67 positive cells while miR-302d-3p increased it. Members of the miR-302 family have been associated with embryonic development, particularly cardiac differentiation [41], pluripotency [42], and cardiomyocytes proliferation [43, 44], mainly by modulation of Hippo signaling. We demonstrated that proliferating cells on D30 were positive for Ki67, despite the absence of EdU incorporation, suggesting the influence of miRNAs at another time in the cell cycle other than DNA synthesis.

Cardiomyocytes maturation may be inferred by the shift of immature proteins isoforms like α-MHC (*MYH6*) and ssTnI (*TNNI1*) to cardiac-specific β-MHC (*MYH7*) and cTnI (*TNNI3*), both in vitro [45, 46] and in vivo [37, 47]. At the same time, sarcomeres alignment and organization are crucial to intracellular organization and signal transduction, thus contributing to cardiomyocytes maturation [48, 49]. We presented evidence that miR-124-3p, miR-1323, and miR-30a-5p promote a shift between *α*-MHC and *β*-MHC on D15, though not sustained for miR-30a-5p on D30. We speculate that this contradiction highlights the permissiveness differences between the effects of Profile1 and Profile2 miRNAs and the complexity of their action in maturation. MiR-124-3p promoted the shift in troponin and myosin heavy chain isoforms along with a decrease in cell proliferation at D30. These results are corroborated by previous findings and may be, at least in part, due to STAT-3 targeting by miR-124-3p [50]. Also, mutated sarcomere protein Titin induces cytoskeleton destabilization that may be counteracted by compensatory increase in miR-124-3p levels [51]. MiR-302d-3p promoted a similar phenotype, which was not replicated by miR-302c-3p. Nevertheless, opposite effects by these members of miR-302 family have been reported previously [44, 52].

Complex Ca^2+^ cycling mechanisms evolve with improvement in contractile performance and sarcomeric organization as maturation occurs [53] and are fine-tuned by miRNAs. MiR-512-3p increased amplitude reflecting in reduced calcium reuptake, due to reduced levels of SERCA2a/b protein and susceptibility to thapsigargin, which is consistent with the fact that miR-512-3p contains sites of interactions with the *ATP2B2* gene (Table 4), responsible for translating one of the cardiac SERCA subunits. For miR-124-3p, the increase in amplitude at first glance may seem indicative of maturation, however, the reduced levels of intracellular Ca^2+^, decreased SR Ca^2+^ release and an abnormal transient after caffeine treatment suggest perturbation of maturation, which may be associated with modifications in Ryanodine Receptor function [54]. MiR-124-3p also increased the decay time (DT 90%) of the calcium transient, which may be explained by miR-124-p binding sites in genes that control calcium efflux, such as ATPases, transient calcium channels, proteins that modulate RYR2 function, Ca^2+^ buffering-associated proteins (Fig. 7A and Table 3) or indirectly by inhibition of potassium channels-related genes as demonstrated by us and elsewhere [50].

**Table 4:** Validated targets of hsa-miR-512-3p associated with cardiac development and maturation aspects

Gene ontology term	Gene symbol
Cardiac muscle cell development	SLC8A1
Maturation aspects	Symbol
Cardiac specification	*MIXL1*
Sodium channel	*SLC8A1*
Potassium channel	*KCNA7*
	*KCNB1*
	*KCND3*
	*KCNJ8*
	*KCNK6*
Calcium channel	*ATP2B2*

The miRNAs evaluated in this study in general had a delaying effect on contractile maturation, particularly by increasing the contraction–relaxation time, and reducing the maximum contraction and relaxation velocities. This response may be linked to different molecular mechanisms related to cardiac maturation including the components responsible for calcium handling [45, 55, 56]. In the context of maturation, it is known that the ssTnI isoform, predominant in immature hiPSC-CMs, has a lower production of tension and greater affinity for Ca^2+^, which reduces the relaxation velocity, as observed in most of the analyzed miRNAs [57, 58]. Interestingly, miR-124-3p was the only one to reduce the contraction speed without changing the relaxation speed. This can be partially explained by the reduction in the expression of the *SCN5A* gene, responsible for increasing the upstroke velocity.

Metabolic maturation of CMs is mainly characterized by a transition in the substrate source, from anaerobic glycolysis in fetal CMs to fatty acid oxidation in adult CMs [59, 60]. Fetal and hiPSC-CMs mitochondria share similar characteristics, such as the reduced number and preferred perinuclear localization [61]. In addition, fetal mitochondria have constitutively open pores, reducing their membrane potential without inducing cytochrome leakage and apoptosis [62]. We demonstrated that miR-302c-3p, miR-302d-3p, and miR-1323 reduced Tom20 levels, whereas only miR-124-3p increased Tom20 and mitochondrial distribution throughout the cytoplasm. In addition, miR-124-3p reduced mitochondrial membrane potential without an expressive increase in apoptosis. We thus suggest that miR-124-3p induces morphological but not functional changes in mitochondria. Interestingly, we found in our bioinformatics analysis that miR-124-3p interacts with *EPM2AIP1*, which is associated with glycogen metabolism [63, 64] and might change mitochondria substrate source to ATP production. We speculate that this may be a potential link between the mitochondrial changes and the hiPSC-CMs maturation, although further experiments are required to test this hypothesis.

The action potential and ion channels are modified throughout the maturation process. MiR-124-3p consistently altered the investigated maturation features and reduced the action potential peak and duration (APD50). We speculate that these effects are associated with prevention of maturation, since the reduction of the gene *SCN5A* leads to slower activation of sodium channels and, therefore, to a lower upstroke velocity. Furthermore, early-stage hiPSC-CMs present an immature isoform of this gene and the miR-124-3p could shift it toward the mature isoform, leading to a lower AP peak. We also observed that 4 of the 8 miR-124-3p target genes influence the plateau phase of the action potential (Fig. 7B) consistent with decreased APD50 observed. Our bioinformatics analyses revealed that miR-124 targets genes associated with sodium, calcium, and potassium channels, which also demonstrates its relevance on hiPSC-CMs’ electrophysiology.

Strategies to stimulate hiPSC-CM maturation include electrical, cyclic strain, fluid flow, and pharmacological approaches [39, 65, 66]. MiRNAs have also been reported to influence maturation. The let-7 mRNA family when overexpressed in differentiated CMs enhances cells contraction force, size, respiratory capacity, and length of the sarcomere [67].

Given the exploratory nature of our work, we must balance these encouraging findings with the fact that we tested two intervals with acute miRNA mimics exposure to validate our data. Considering that the kinetics of our intervention and the differentiation process are not well defined, one may argue that the real modulatory effects remain unknown. It is accurate, however, that the circumstantial evidence also points to further exploration of miRNA/target genes interactions. We recognize that to properly assess the role of Profile 2 miRs 532-5p and 30a-5p, the use of inhibitors instead of mimics could be a more informative approach. Also, our study was focused on hiPSC-CM cultured on a bidimensional (2D) system, which likely undermines the complexity of the cardiac microenvironment. On that matter, both hiPSC-CM contact with supporting cells like fibroblasts and endothelial cells [68], and the surrounding extracellular matrix [69] were already shown to potentiate hiPSC-CM maturation and differentiation. Further studies would then benefit from evaluating the miRNAs presented here on a more in vivo-like environment. Finally, the global gene expression comparisons with cardiac samples, to demonstrate the in vivo relevance of these findings are limited by the fact that tissues comprise a wide variety of cell types that could mask or interfere with the results.

## Conclusions

The results presented here demonstrate that most of the selected miRNAs influence the cardiac proliferation–maturation axis corroborating the in silico analysis. The miRNAs exhibiting Profile 1 when overexpressed delayed maturation characteristics mainly associated with structural changes, whereas miR-124-3p regulated both structural and functional features. Altogether, we (1) identified a molecular signature for tracking in vitro cardiac differentiation over time; (2) provided evidence that the miRNA/gene pairs display biological relevance; and (3) showed that most of the top-scoring miRNAs influence the proliferation–maturation axis. The improved understanding of the interplay among miRNAs and gene targets may be instrumental in developing more robust human cardiac disease modeling systems and cardiac cell-based regenerative approaches.

## Supplementary Information

**Additional file 1:** Supplementary Table S1.

**Additional file 2:** Figures S1–S5 and Supplementary Tables S2–S3.

## Data Availability

All data generated during this study and supplementary files, tables, and methods are included in this article. Databases from the GEO repository (https://www.ncbi.nlm.nih.gov/geo/) are identified by their GSE ID and assessed in the GEO datasets details spreadsheet in the.

## Abbreviations

APD50: Action potential duration to 50%
ASPH: Aspartate beta-hydroxylase
ATP1A1: ATPase Na^+^/K^+^ transporting subunit alpha 1
ATP2B2: ATPase sarcoplasmic/endoplasmic reticulum Ca^2+^ transporting 2
ATP2B4: ATPase plasma membrane Ca^2+^ transporting 4
AUC: Area under the curve
BSA: Bovine serum albumin
CACNA1C: Calcium voltage-gated channel subunit alpha1 C
CACNA2D1: Calcium voltage-gated channel auxiliary subunit alpha2/delta 1
CACNB3: Calcium voltage-gated channel auxiliary subunit beta 3
[Ca^2+^]i: Intracellular Ca2^+^ concentration
CALM1: Calmodulin 1
CALM3: Calmodulin 3
CAMK2D: Calcium/calmodulin-dependent protein kinase II Delta
CASQ2: Calsequestrin 2 (cardiac muscle)
CCNB1: Cyclin B1
CHIR99021: GSK-3 inhibitor
cTNI: Cardiac troponin I—gene TNNI3
Ctrl: Control
D15: Day 15 of differentiation
D23: Day 23 of differentiation
D30: Day 30 of differentiation
DAPI: 4′,6-Diamidino-2-phenylindole
DE: Differential expression
DT90: Time until 90% decay
EdU: 5-Ethynyl-2′-deoxyuridine
EPM2AIP1: EPM2A (Laforin) interacting protein 1
FKBP1A: FKBP prolyl isomerase 1A
GO: Gene ontology
HCN4: Hyperpolarization-activated cyclic nucleotide-gated potassium channel 4
hESCs: Human embryonic stem cells
hiPSC-CM: Human-induced pluripotent stem cell-derived cardiomyocytes
iFC: Inverse fold-change
KCNA1: Potassium voltage-gated channel subfamily A member 1
KCNA5: Potassium voltage-gated channel subfamily A member 5
KCNE1: Potassium voltage-gated channel subfamily E regulatory subunit 1
KCNE4: Potassium voltage-gated channel subfamily E regulatory subunit 4
KCNJ2: Potassium inwardly rectifying channel subfamily J member 2
LBH: Limb-bud and heart
MICB: MHC class I polypeptide-related sequence B
miRNAs: MicroRNAs
OCT4: Octamer-binding protein 4
PCA: Principal component analysis
PFA: Paraformaldehyde
ROC: Receiver operating characteristic
RYR2: Ryanodine receptor 2
SCN4B: Sodium voltage-gated channel beta subunit 4
SCN5: Sodium voltage-gated channel alpha subunit 5
SERCA 2 a/b: ATPase sarcoplasmic/endoplasmic reticulum Ca^2+^ transporting 2
ssTnI: Slow skeletal troponin I—gene TNNI1
TAU: Decay time constant
TMEM30B: Transmembrane protein 30B (cell cycle control protein 50B)
Tom20: Translocase of outer mitochondrial membrane 20
TROPO-GFP: HiPSC line mono-allelic mEGFP-tagged TNNI1 WTC
TSPAN33: Tetraspanin 33
WNT: Wnt/*β*-Catenin signaling pathway
*α*-MHC: Myosin heavy chain 6—gene *MYH6*
*β*-MHC: Myosin heavy chain 7—gene *MYH7*
