# Supplementary material for: Time-regulated transcripts with the potential to modulate human pluripotent stem cell-derived cardiomyocyte differentiation
Source: Stem Cell Res Ther. 2022 Sep 2;13:437. doi: 10.1186/s13287-022-03138-x (PMC9438174; doi:10.1186/s13287-022-03138-x)
Supplement: Supplementary file 2 — Additional file 2: Figures S1–S5 and Supplementary Tables S2–S3. [file 13287_2022_3138_MOESM2_ESM.docx]

**Additional file 2.**


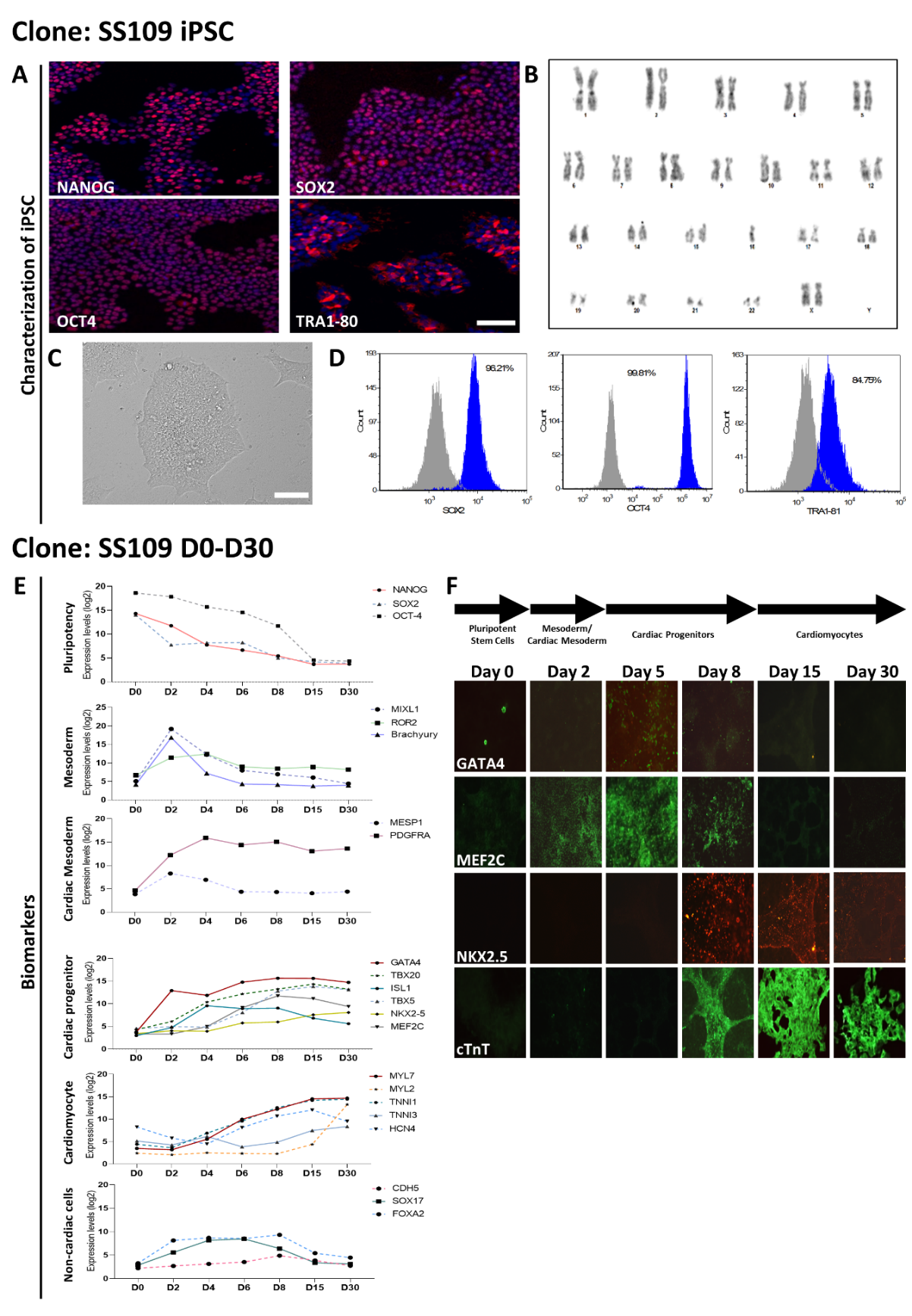


**Figure S1:** Characterization, morphological and molecular profiling of SS109 clone. **A** Photomicrographs of immunofluorescence staining for pluripotency markers (NANOG, SOX2, OCT4, TRA1-80; red) and nuclei count-stained with DAPI (blue); scale bar = 50 μm. **B** The analyzed cell line shows normal karyotype. **C** Bright field photomicrograph of hiPSC colony displaying typical morphology. **D** Representative overlay histograms of expression of pluripotency markers SOX-2, OCT-4 and TRA1-80. Gray curve represents negative control and blue curve represents test sample. **E** Biomarkers gene expression for pluripotency, mesoderm, cardiac mesoderm, cardiac progenitor, cardiomyocytes, and non-cardiac cells of different stages of hiPSC-CM differentiation were analyzed by microarray of samples from days 0 to 30 (N = 3 independent differentiation experiments). **F** Representative immunostainings of GATA4, MEF2c, NKX2.5, and cTnT at different stages of differentiation.


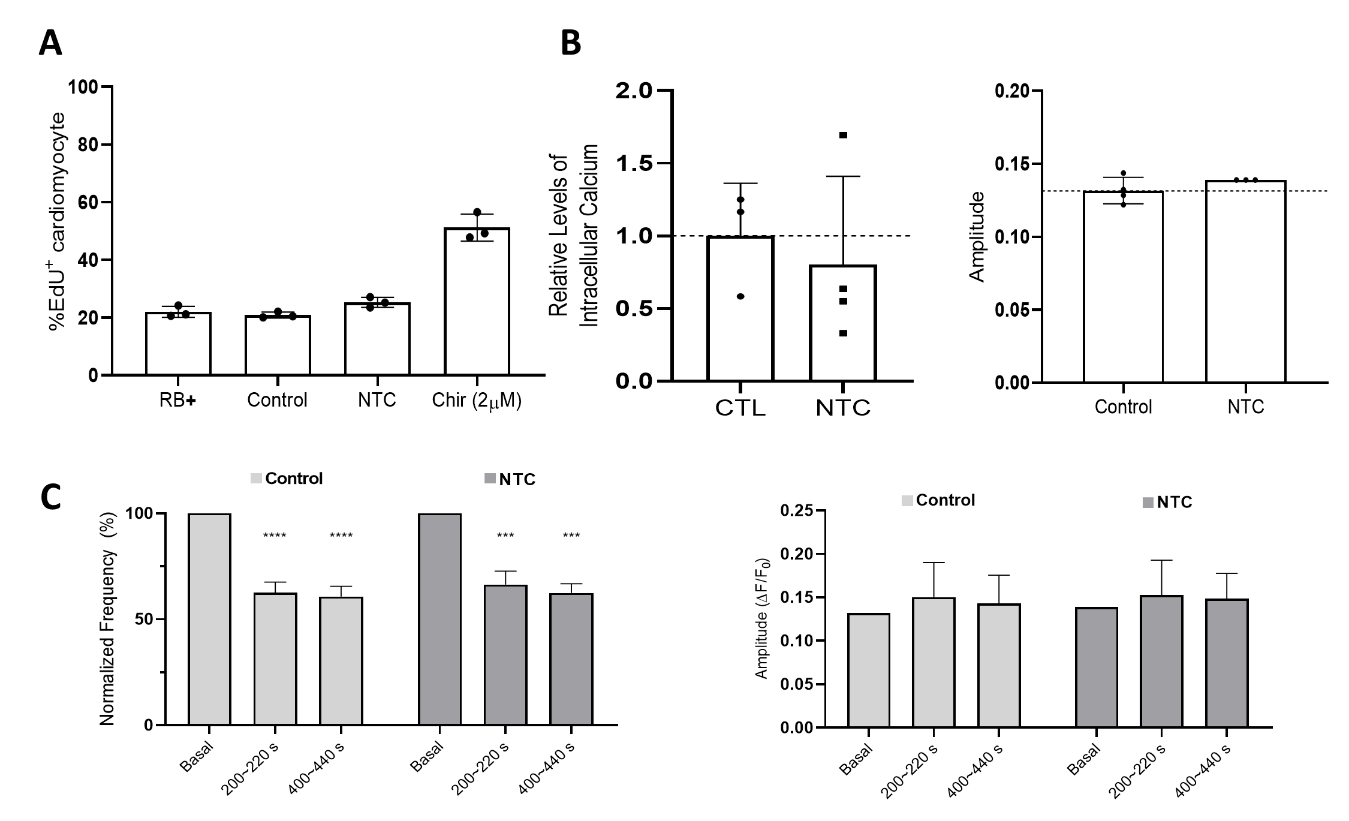


**Figure S2:** Validation of negative control of miRNAs transfections. **A** EdU incorporation. **B** Relative levels of intracellular calcium and amplitude. **C** Effects of SERCA inhibitor thapsigargin (5µm) on calcium frequency and amplitude in three-point: Basal (20 s), 200~220 s and 400~440 s in spontaneously beating cells. Normalized frequency (%) displayed as percentage decreased versus baseline condition) (N = 3-4 independent differentiation experiments).


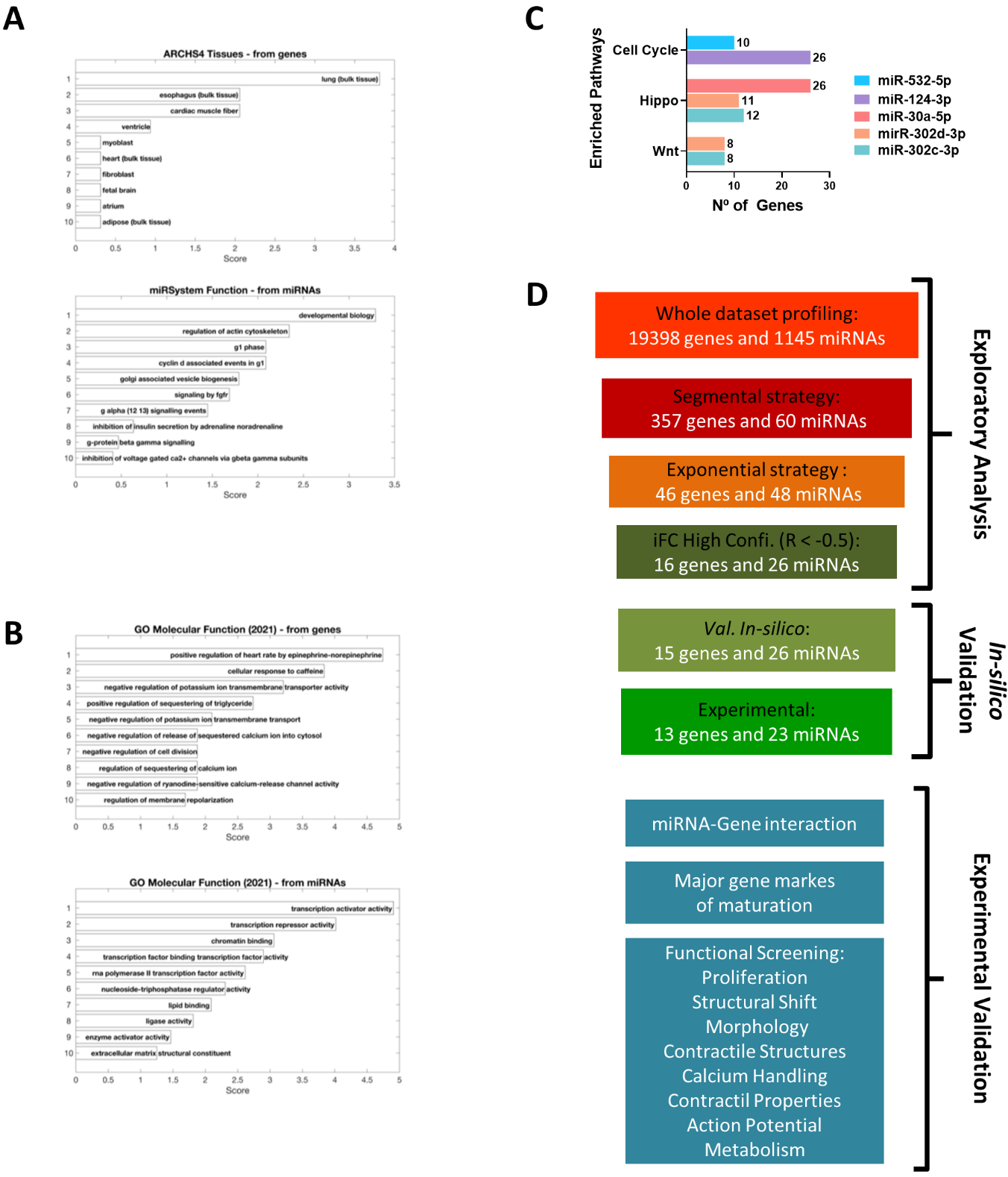


**Figure S3:** Gene ontology analysis and schematic workflow. **A** ARCHS4 tissue annotation showing cardiac related genes in our filtered transcripts and miRSystem Function depicting the closer relationship of the miRNAs obtained in our selection approach with cell-cycle regulation and developmental biology. **B** GO molecular function for the list of transcripts showed several enrichments associated with the cardiac handling of Calcium and the miRNA pathways enriched for high transcriptional activity. **C** Bar graph of selected miRNAs with Pearson’s R < -0.5 and p < 0.01 subjected to TarBasev7.0 GO analysis and its experimentally validated targets. In this analysis, five of eight miRNAs displayed genes associated with proliferation pathways. **D** Workflow of this study with steps performed in exploratory analysis followed by *in-silico* and experimental validation.


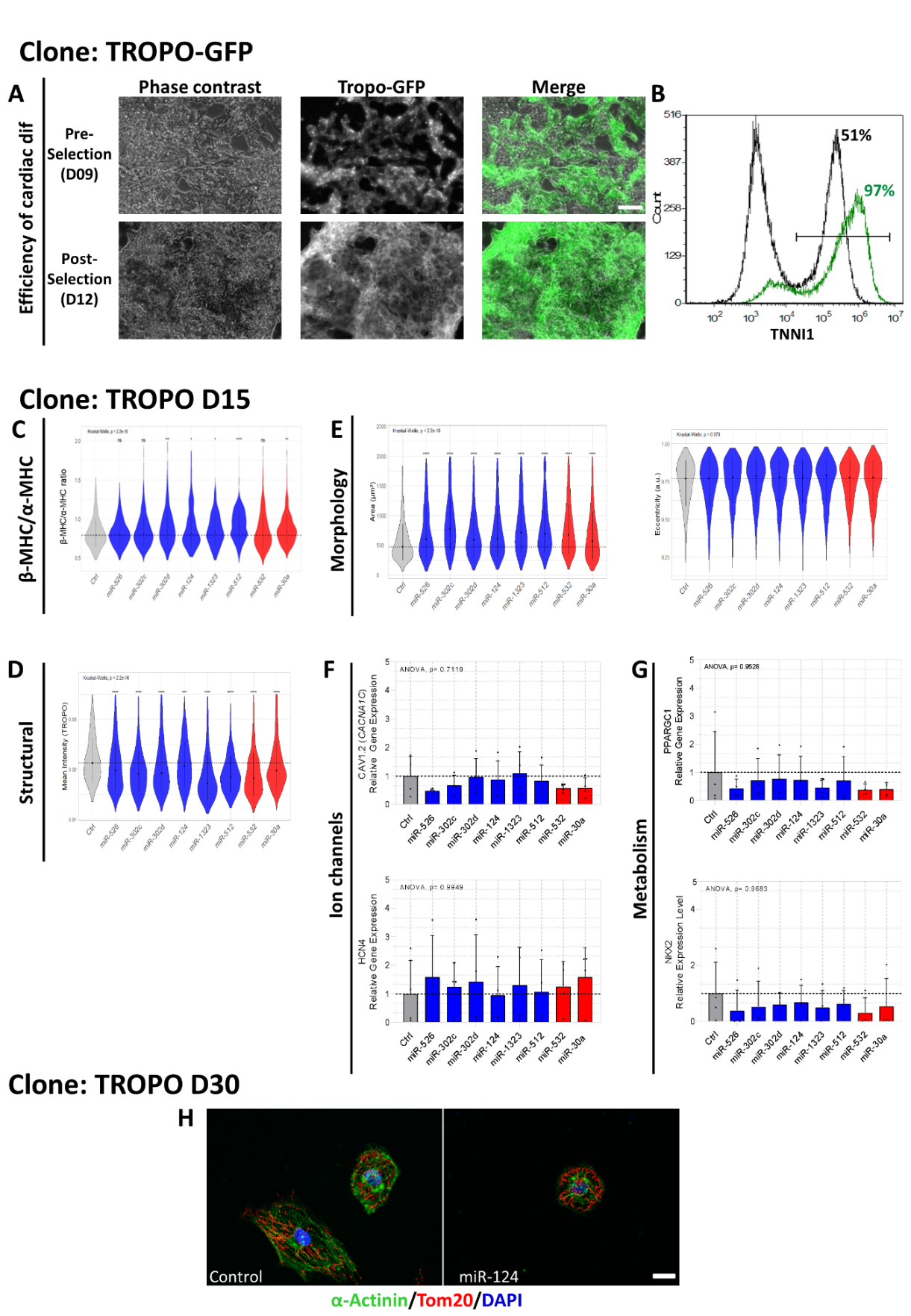


**Figure S4.** Characterization, morphological and molecular profiling of TROPO-GFP hiPSC-CMs at D15 and D30 of differentiation. **A** Phase contrast and TROPO-GFP photomicrographs of hiPSC-CMs at D09 (pre-metabolic selection) and D12 (post-metabolic selection) showing the efficiency of cardiac differentiation. **B** Overlay histograms of expression of TNNI1 by hiPSC-CMs. Black and green curves represent hiPSC-CM at D09 and D12, respectively. Violin plots MYH7/MYH6 ratio relative to Figure 3F (**C**) troponin mean fluorescence intensity (**D**), cell area and eccentricity (**E**). Box plots presenting gene expression levels of the ion channels Cav1.2 and HCN4 (**F**), and metabolism-related markers PPARGC1 NKX2 (**G**). **H** Representative immunostainings α-Actinin, Tom20 and DAPI (scale bar = 20 µm). (N = 4 independent differentiation experiments). Dashed line represents the median of control cells in each graph. Statistical significance treatment vs control (Ctrl) are represented as * p < 0.05, ** p < 0.001, *** p < 0.001.


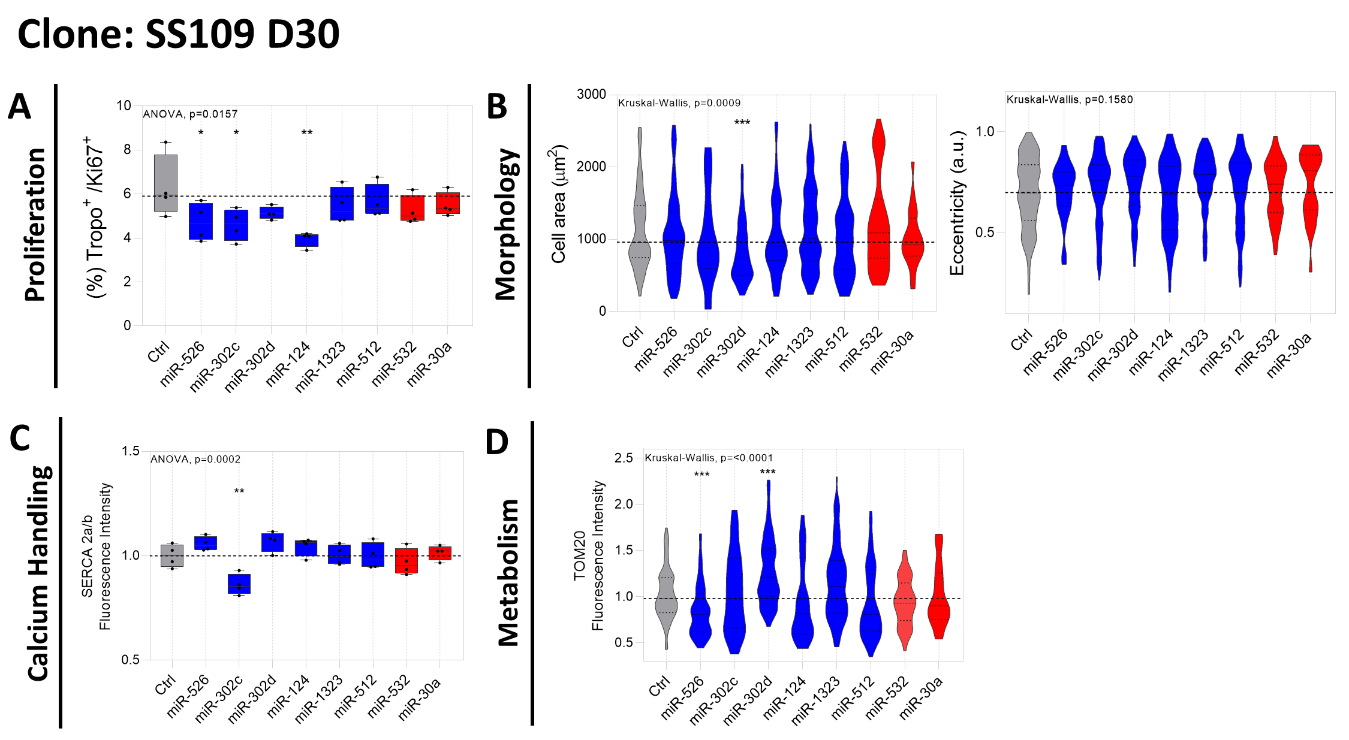


**Figure S5: A** Box plot presenting percentage of cells positive for both troponin and Ki67 among troponin positive cells of SS109 clone. **B** Violin plots presenting cell area (**left**) and eccentricity (**right**). **C** Box plot presenting gene expression levels of SERCA2a/b. **D** Violin plot presenting Tom20 mean fluorescence intensity. Dashed line represents the median of control cells in each graph. (N = 3-4 independent differentiation experiments). Statistical significance treatment vs control (Ctrl) are represented as * p < 0.05, ** p < 0.001, *** p < 0.001.

**Supplementary Table S2:** Oligonucleotide sequences

| **miRNA**  **Nome** | **miRBase**  **accession n.** | **Sequence- seed region (targetScanHuman)** |
| --- | --- | --- |
| hsa-miR-124-3p | MIMAT0000422 | 5´- UAAGGCACGCGGUGAAUGCCAA – 3´ |
| hsa-miR-512-3p | MIMAT0002823 | 5´- AAGUGCUGUCAUAGCUGAGGUC – 3´ |
| hsa-miR-302d-3p | MIMAT0000718 | 5´- UAAGUGCUUCCAUGUUUGAGUGU – 3´ |
| hsa-miR-1323 | MIMAT0005795 | 5´- UCAAAACUGAGGGGCAUUUUCU – 3´ |
| hsa-miR-532-5p | MIMAT0002888 | 5´- CAUGCCUUGAGUGUAGGACCGU – 3´ |
| hsa-miR-526b | MIMAT0002835 | 5´- CUCUUGAGGGAAGCACUUUCUGU– 3´ |
| hsa-miR-30a-5p | MIMAT0000087 | 5´- UGUAAACAUCCUCGACUGGAAG – 3´ |
| hsa-miR-302c-3p | MIMAT0000717 | 5´- UAAGUGCUUCCAUGUUUCAGUGG – 3´ |

**Supplementary Table S3:** Primers used for qRT-PCR

| **Gene** | **Sense Primer (5’-3’)** | **Antisense Primer (5’-3’)** |
| --- | --- | --- |
| EPM2AIP1 | GGGTGACTTCGTTCACCAGT | GTCCCTGGCTCGGTTAAACA |
| LBH | CTATCAGATCTTTCCCGACCCA | GTTCCACCACTATGGAGGGC |
| CASQ2 | GGTGGAATTTCTCTTGGACCTCA | ACGCTTTGTAATATTCTGAGTCCT |
| MICB | GGAATGGAACCTACCAGACCTG | CTGTCCGTTGACTCTGAAGCAC |
| TSPAN33 | CAGAAGAAGTTCAGCTGCTGC | GCTTCCAAGTAGTCCAGAGCC |
| TMEM30B | GGCCCCGTGTACCTCTACTA | GTCGGAGCTGAACTGGTAGG |
| KCNJ2 | AACAGTGCAGGAGCCGCTTTGT | AGGACGAAAGCCAGGCAGAAGA |
| SCN5a | CAAGACCTGCTACCACATCGTG | GTCGGCATACTCAAGCAGAACC |
| CACNA1C | GCAGGAGTACAAGAACTGTGAGC | CGAAGTAGGTGGAGTTGACCAC |
| RYR2 | CAAGGCAGCTCTGGACTTCA | CCAGGCTAGGTAGAGGAAGGA |
| ATP2A2 | GGACTTTGAAGGCGTGGATTGTG | CTCAGCAAGGACTGGTTTTCGG |
| HCN4 | CAACCCGGGGTCAACAAATTC | AGGTTTCCCACCATCAGCAG |
| GAPDH | GTCTCCTCTGACTTCAACAGCG | ACCACCCTGTTGCTGTAGCCAA |
